# Out of and in East Asia: phylogeny, biogeography and diversification of Thalictroideae (Ranunculaceae) in the Northern Hemisphere

**DOI:** 10.1093/aob/mcae148

**Published:** 2024-08-28

**Authors:** Yuan-Yuan Ling, Huan-Wen Peng, Lian Lian, Andrey S Erst, Kun-Li Xiang, Wei Wang

**Affiliations:** State Key Laboratory of Plant Diversity and Specialty Crops, Institute of Botany, Chinese Academy of Sciences, Beijing 100093, China; China National Botanical Garden, Beijing 100093, China; University of Chinese Academy of Sciences, Beijing 100049, China; State Key Laboratory of Plant Diversity and Specialty Crops, Institute of Botany, Chinese Academy of Sciences, Beijing 100093, China; China National Botanical Garden, Beijing 100093, China; University of Chinese Academy of Sciences, Beijing 100049, China; State Key Laboratory of Plant Diversity and Specialty Crops, Institute of Botany, Chinese Academy of Sciences, Beijing 100093, China; China National Botanical Garden, Beijing 100093, China; Central Siberian Botanical Garden, Siberian Branch of Russian Academy of Sciences, Zolotodolinskaya str. 101, Novosibirsk 630090, Russia; State Key Laboratory of Plant Diversity and Specialty Crops, Institute of Botany, Chinese Academy of Sciences, Beijing 100093, China; China National Botanical Garden, Beijing 100093, China; State Key Laboratory of Plant Diversity and Specialty Crops, Institute of Botany, Chinese Academy of Sciences, Beijing 100093, China; China National Botanical Garden, Beijing 100093, China; University of Chinese Academy of Sciences, Beijing 100049, China

**Keywords:** Biogeography, East Asia, Miocene, molecular dating, phylogeny, Thalictroideae

## Abstract

**Background and Aims:**

Understanding the biogeographical patterns and processes underlying the distribution of diversity within the Northern Hemisphere has fascinated botanists and biogeographers for over a century. However, as a well-known centre of species diversity in the Northern Hemisphere, whether East Asia acted as a source and/or a sink of plant diversity of the Northern Hemisphere remains unclear. Here, we used Thalictroideae, a subfamily widely distributed in the Northern Hemisphere with the majority of species in East Asia, to investigate the role of East Asia in shaping the biogeographical patterns of the Northern Hemisphere and to test whether East Asia acted as a museum or a cradle for herbaceous taxa.

**Methods:**

Based on six plastid and one nuclear DNA regions, we generated the most comprehensive phylogeny for Thalictroideae, including 217 taxa (~66 % species) from all ten of the currently recognized genera. Within this phylogenetic framework, we then estimated divergence times, ancestral ranges and diversification rates.

**Key Results:**

The monophyletic Thalictroideae contains three major clades. All genera with more than one species are strongly supported as monophyletic except for *Isopyrum*, which is nested in *Enemion*. The most recent common ancestor of Thalictroideae occurred in East Asia in the late Eocene (~36 Mya). From the Miocene onwards, ≥46 dispersal events were inferred to be responsible for the current distribution of this subfamily. East Asian Thalictroideae lineages experienced a rapid accumulation at ~10 Mya.

**Conclusions:**

The biogeographical patterns of Thalictroideae support the ‘out of and in East Asia’ hypothesis, i.e. East Asia is both a source and a sink of biodiversity of the Northern Hemisphere. The global cooling after the middle Miocene Climatic Optimum, combined with the exposed land bridges owing to sea-level decline, might jointly have caused the bidirectional plant exchanges between East Asia and other Northern Hemisphere regions. East Asia serves as evolutionary museums and cradles for the diversity of Thalictroideae and probably for other herbaceous lineages.

## INTRODUCTION

The biogeographical distributions of plants in the Northern Hemisphere have fascinated botanists and biogeographers for over a century (Wolfe, 1975; Donoghue *et al.*, 2001; Xiang and Soltis, 2001). Numerous plant groups in the Northern Hemisphere have been reported to exhibit intercontinental disjunct distributions involving East Asia, West Asia, Europe and North America (Wolfe, 1975; Donoghue *et al.*, 2001; Milne and Abbott, 2002). Biogeographical studies using the integration of phylogenetic, molecular dating and biogeographical methods have largely focused on the disjunct distribution between East Asia and North America (e.g. Wen *et al.*, 2010; Xiang *et al.*, 2015; Kim *et al.*, 2019; Chen *et al.*, 2020; Peng *et al.*, 2023). A few studies have also been devoted to investigate the Europe–North America disjunction (Fuselier *et al.*, 2009; Kadereit and Baldwin, 2012). However, historical biogeography of the whole Northern Hemisphere remains incompletely understood.

It is well known that species diversity is very unevenly distributed in the Northern Hemisphere (Myers *et al.*, 2000). In comparison to other Northern Hemisphere regions, East Asia harbours exceptional biodiversity and endemics, containing >20 000 species of seed plants and >600 endemic genera (Wu and Wu, 1996; Qian, 2002; Manchester *et al.*, 2009), which makes it one of the biodiversity hotspots for seed plants (Xing and Ree, 2017; Lu *et al.*, 2018). For most plant taxa with East Asian–North American disjunction, East Asia is usually recognized as the primary source for species diversity (Wen, 1999; Milne and Abbott, 2002; Deng *et al.*, 2015). The East Asian––Tethyan disjunct *Quercus* section *Ilex* also originated in East Asia (Jiang *et al.*, 2019). East Asia has also been suggested as a sink for the diversity of some genera, with immigrants from Europe (Tu *et al.*, 2010), North America (Aradhya *et al.*, 2007; Chen *et al.*, 2020) or topical Asia (Thomas *et al.*, 2012; Xiao *et al.*, 2022). Meanwhile, a few studies show that floristic exchanges might be bidirectional between East Asia and Europe or between East Asia and North America (Yoo *et al.*, 2018; Zhou *et al.*, 2020). Thus, biogeographical relationships between East Asia and other Northern Hemisphere regions are complex. To better understand the role of East Asia in the formation of the Northern Hemisphere biodiversity, one needs to examine the biogeographical patterns of the Northern Hemisphere in a broader phylogenetic context.

Additionally, the high present-day species diversity found in an ecoregion or biome is usually portrayed as a consequence of the gradual accumulation or preservation of species over time (Stebbins, 1974; Gaston and Blackburn, 1996) or as a relatively recent and rapid speciation (Haffer, 1969; Richardson *et al.*, 2001). These two scenarios correspond to the ‘museum’ and ‘cradle’ hypotheses, respectively (McKenna and Farrell, 2006; Moreau and Bell, 2013; Wiens, 2017). A genus-level study suggests that East Asia might be a museum for herbaceous taxa (Lu *et al.*, 2018). Yet, at the species level, whether East Asia is still a museum or a cradle for herbaceous taxa remains elusive.

The subfamily Thalictroideae (Ranunculaceae) comprises ten genera with ~320 herbaceous species (Tamura, 1995; Wang and Chen, 2007), including many taxa of pharmaceutical and horticultural interest, such as *Aquilegia coerulea*, *Dichocarpum dalzielii* and *Thalictrum grandiflorum*, with *Aquilegia* as a model system for studying plant development, ecology and evolution (Kramer, 2009; Sharma *et al.*, 2019; Singh *et al.*, 2020). This subfamily is widely distributed in the Northern Hemisphere, with its centre of diversity in East Asia (Tamura, 1995; Wang and Chen, 2007). *Thalictrum* (~200 spp.) and *Aquilegia* (~80 spp.) are the two largest genera in Thalictroideae and are widely distributed in the Northern Hemisphere, with several species of *Thalictrum* extending to Southern America and Africa. *Enemion* (six spp.) exhibits a disjunct distribution between East Asia and North America. The monotypic *Leptopyrum* is endemic to northern and eastern Asia. *Isopyrum* (three spp.). *Paropyrum* (one sp.), and *Paraquilegia* (five spp.) are distributed in western China, central and northern Asia, and southern Europe. The remaining three genera, *Dichocarpum* (20 spp.), *Semiaquilegia* (three spp.) and *Urophysa* (two spp.), are endemic to East Asia. Thalictroideae is characterized by a high level of regional endemism, and East Asia is the richest in the numbers of the genera, species and endemic species of the subfamily (Hsiao, 1979; Tamura, 1995). Thus, Thalictroideae not only provides an opportunity to investigate the biogeographical relationships between East Asia and other Northern Hemisphere regions, but also serves as an ideal model to test whether the museum or cradle hypothesis can explain the diversification patterns of herbaceous taxa in East Asia.

Previous phylogenetic studies have greatly improved our understanding of the intergeneric relationships of Thalictroideae (Wang and Chen, 2007; Cossard *et al.*, 2016; Wang *et al.*, 2016; Zhai *et al.*, 2019). Of the ~320 species, <15 species have been sampled in these studies. Meanwhile, there are some molecular dating and biogeographical studies focusing on several genera of this subfamily, such as *Aquilegia* (Fior et *al.*, 2013) and *Dichocarpum* (Xiang *et al.*, 2017). However, the biogeographical origin and diversification process of Thalictroideae remain unknown to date.

In this study, our objectives were to reconstruct a species-level phylogeny for Thalictroideae with the most comprehensive taxon sampling to date, and thereafter to investigate the spatiotemporal evolution of the subfamily. In particular, we ask:

(1) Did East Asia serve as a source area for the Northern Hemisphere diversity in Thalictroideae, and when did the major dispersal events occur?
(2) Does the museum or cradle hypothesis prevail for Thalictroideae in East Asia?

## MATERIALS AND METHODS

### Taxon sampling and data assembly

We sampled 217 taxa of Thalictroideae (Ranunculaceae) representing ~66 % extant Thalictroideae species and covering all ten genera recognized by Wang and Chen (2007). Our sampling includes 107 species of *Thalictrum* and 71 species of *Aquilegia*, representing 54 and 89 % of species diversity of the two largest genera in Thalictroideae, respectively. One species of Ranunculoideae (*Calathodes oxycarpa*) and three species of Coptidoideae (*Coptis chinensis*, *Coptis trifolia* and *Xanthorhiza simplicissima*) were selected as outgroups, of which Coptidoideae was used to root the tree based on our previous studies (Wang *et al.*, 2009, 2016). Voucher information and GenBank accession numbers are listed in Supplementary Data (Table S1).

Jian *et al.* (2008) suggested that a combination of relatively slowly evolving and rapidly evolving DNA regions can provide good resolution for both deep-level and shallow-level relationships. In this study, we selected six plastid (*rbcL*, *matK*, *ndhA*, *trnL*-*F*, *atpB*-*rbcL* and *rpl32*-*trnL*) and one nuclear (ITS) DNA region, among which *rbcL* and *matK* have been used to reconstruct the genus-level relationships of Thalictroideae (Wang and Chen, 2007), whereas the other five have mainly been used for the species-level relationships within Ranunculaceae (Jiang *et al.*, 2017; Xiang *et al.*, 2017; Kadereit *et al.*, 2019). Genomic DNA extraction, PCR amplification and sequencing followed Wang *et al.* (2017). The primers used in this study are shown in Supplementary Data (Table S2). A total of 602 new sequences from 107 taxa were generated: *rbcL*, 85; *matK*, 74; *ndhA*, 94; *trnL*-*F*, 85; *atpB*-*rbcL*, 103; *rpl32*-*trnL*, 84; and ITS, 77. Sequences were aligned in Geneious v.9.0.2 (Kearse *et al.*, 2012), then adjusted manually. Four difficult-to-align regions in *ndhA* (encompassing 85 sites), *trnL*-*F* (encompassing 77 sites) and *rpl32*-*trnL* (encompassing 103 sites) were excluded from all analyses.

### Phylogenetic analyses

We initially used maximum likelihood (ML) method to conduct non-parametric bootstrap (BS) analyses for each plastid locus in RAxML v.8.2.12 (Stamatakis, 2014). No significant conflicting node (BS values ≥ 70 %) was detected among individual plastid loci; consequently, six plastid matrices were combined to be the plastid dataset. Phylogenetic analyses for the plastid, ITS and combined plastid and ITS datasets were performed using ML and Bayesian inference (BI) methods in RAxML v.8.2.12 and MrBayes v.3.2.7 (Ronquist *et al.*, 2012), respectively. RAxML was carried out using the GTR+G substitution model for each DNA region and the fast bootstrap option with 1000 replicates. For BI analyses, each DNA region was assigned its own model of nucleotide substitution, as determined by the Akaike information criterion (AIC) via PartitionFinder v.2.1.1 (Lanfear *et al.*, 2016). Two independent runs, each consisting of four Markov chain Monte Carlo (MCMC) chains, were conducted, sampling one tree every 1000 generations over 20 million generations. After assessing the stationarity with Tracer v.1.7.1 (Rambaut *et al.*, 2018), the first 25 % of sampled trees were removed as burn-in and the remaining trees were used to construct a majority rule (>50 %) consensus tree. To assess the combinability between the plastid and ITS datasets, we adopted the tree-based comparison method of Wiens (1998) and chose the thresholds BS ≥ 70 % and posterior probability (PP) ≥ 0.95 as an indication of strongly supported incongruence, following the suggestion of Wang *et al.* (2014*b*).

### Divergence time estimation

Divergence times were estimated using the combined plastid and ITS dataset in BEAST v.2.3.0 (Bouckaert *et al.*, 2014). We used a reduced dataset of 148 species of Thalictroideae that excluded species with zero branch lengths in *Aquilegia* and species with extremely broad geographical distribution in *Thalictrum*. Considering that there are no reliable Thalictroideae fossils, we selected three secondary calibration points with a normal prior distribution, taking ages estimated in a broader study of Ranunculaceae (Wang *et al.*, 2016). The stem and crown group ages of Thalictroideae were constrained to 82.78 (s.d. = 3.1) and 33.81 (s.d. = 4.0) Mya, respectively. The maximum age of the root was set at 89.9 (s.d. = 4.0) Mya, which is the estimated stem group age of Coptidoideae.

We first used Bayes factors (Kass and Raftery, 1995), calculated by marginal likelihoods derived from path sampling (Baele *et al.*, 2013), to compare four clock models (strict, exponential, lognormal and random). The lognormal clock model was optimal for our data (Supplementary Data Table S3). We also used Bayes factors to compare two distinct tree prior processes: yule and birth–death. A birth–death tree prior was identified as optimal for our data (Supplementary Data Table S4). Under the optimal clock model and tree prior, the BEAST analysis was run under the GTR+I+Γ model for each DNA region separately. The MCMC chains were run for 100 million generations, sampling every 20 000 generations. Convergence and adequate effective sample size values (>200) were checked in Tracer v.1.7.1 (Rambaut *et al.*, 2018). After discarding the initial 25 % sampled trees as burn-in, the maximum clade credibility (MCC) tree, with the mean age and 95 % highest posterior density (HPD) interval for each node, was calculated using TreeAnnotator v.2.3.0 (Bouckaert *et al.*, 2014).

### Ancestral range estimation

Based on the floristic divisions (Takhtajan, 1986; Wu and Wu, 1996) and the present distributions of Thalictroideae species, we defined nine geographical areas: (A) Europe; (B) North Asia; (C) Qinghai–Tibet Plateau (QTP); (D) East Asia; (E) Japan; (F) North America; (G) Africa; (H) Tropical Asia; and (I) South America. Owing to the challenges in scoring under-sampled outgroups for geographical areas, ancestral range estimation was restricted to Thalictroideae. Owing to limited species included in the MCC tree, *Aquilegia* was regarded as an operational taxonomic unit and was coded as East Asia and Japan for its distribution based on the results of Fior *et al.* (2013). Based on weighted AIC corrected for sample size (AICc_wt) using an ML framework, we evaluated four biogeographical models that accommodate vicariance: DEC, DEC+*j*, DIVALIKE and DIVALIKE+*j*. With the highest AICc_wt value, the DEC+*j* model outperformed other models (Supplementary Data Table S5) and was used to infer ancestral ranges. The founder-event speciation parameter (*j*) was criticized (Ree and Sanmartín, 2018), but was recently defended (Matzke, 2022). For comparison, we also run an analysis under the DEC model, which outperformed the DIVALIKE model for our data. To account for phylogenetic uncertainties, we estimated ancestral ranges using the statistical DEC+*j* or DEC model in BioGeoBEARS (Matzke, 2014), as implemented in RASP v.4.2 (Yu *et al.*, 2020). The MCC tree and 1000 posterior trees sampled from the BEAST analysis were loaded as input files. According to geological changes in the Cenozoic (McKenna, 1983; Scotese, 2001; Brikiatis, 2014) and previous biogeographical studies on plants with similar regions (Ebersbach *et al.*, 2017), we specified dispersal probabilities between geographical areas for three separate time slices (Supplementary Data Table S6). Referring to Ebersbach *et al.* (2017), we calculated the cumulative probabilities for estimated ancestral ranges.

### Ancestral habitat reconstruction

Ancestral habitat reconstruction was performed using the Bayesian binary MCMC (BBM) method implemented in RASP v.4.2 (Yu *et al.*, 2020). The MCC tree and 1000 posterior trees generated in the BEAST analysis but excluding outgroups were loaded as input files. Two states were coded for habitat type based on light requirements: closed (forest understorey with at least a partial canopy) and open (without a canopy of other plants). *Aquilegia* was regarded as an operational taxonomic unit and was coded as closed + open based on the results of Bastida *et al.* (2010). We applied ten simultaneous chains optimized with the fixed JC+G model for 5 million generations and sampled the posterior distribution every 1000 generations.

### Diversification rate analyses

The temporal dynamics of diversification rates of Thalictroideae were investigated using three methods with the MCC tree from which all outgroups were pruned. First, we used Bayesian analysis of macroevolutionary mixtures (BAMM) implemented in BAMM v.2.5 (Rabosky, 2014) to infer speciation and extinction rates, in addition to the possible shifts of speciation across the phylogeny. Missing taxa were taken into account by assigning clade-specific sampling fractions (for details, see Supplementary Data Table S7). We used the setBAMMpriors function in BAMMtools v.2.1.6 (Rabosky *et al.*, 2014) to estimate appropriate prior values for BAMM. Four independent reversible jump MCMC chains of 10 million generations were run, sampling parameters every 1000 generations. Chain convergence was checked by calculating the effective sample size values (>200) of the log-likelihood and number of shifts in the R package CODA v.0.19.3 (Plummer *et al.*, 2006). After discarding the first 25 % samples as burn-in, we used BAMMtools to summarize rates over each branch of the phylogeny (phylorate) and plot the best shift configuration with the maximum *a posteriori* probability. We also used the plotRateThroughTime function in BAMMtools to plot speciation, extinction and net diversification rates. Second, we explored regional diversification of Thalictroideae using the nodal ‘lineage density’ method (Mahler *et al.*, 2010). Lineage accumulation within the nine geographical areas was visualized in the R package phytools v.0.7.47 (Revell, 2012). Finally, we used the geographical state-dependent speciation and extinction (GeoSSE) model (Goldberg *et al.*, 2011), as implemented in the R package diversitree (Fitzjohn, 2012), to examine whether the diversification rate in East Asia is higher than that in other regions (East Asia vs. non-East Asia). To correct incomplete sampling biases, we applied state-specific sampling fractions (East Asia, 0.42; non-East Asia, 0.30; both, 0.88). We tested five models (Supplementary Data Table S8), and the fit for the different models was evaluated using the AIC values. To obtain the posterior distributions of the parameters, we conducted a Bayesian GeoSSE analysis under the best-fitting model (no speciation between regions; Supplementary Data Table S8). This was run for 100 000 generations using an exponential prior with a rate of 1/(2*r*), where *r* is the diversification rate of the character (FitzJohn *et al.*, 2009).

## RESULTS

### Phylogeny

**Fig. 1.**
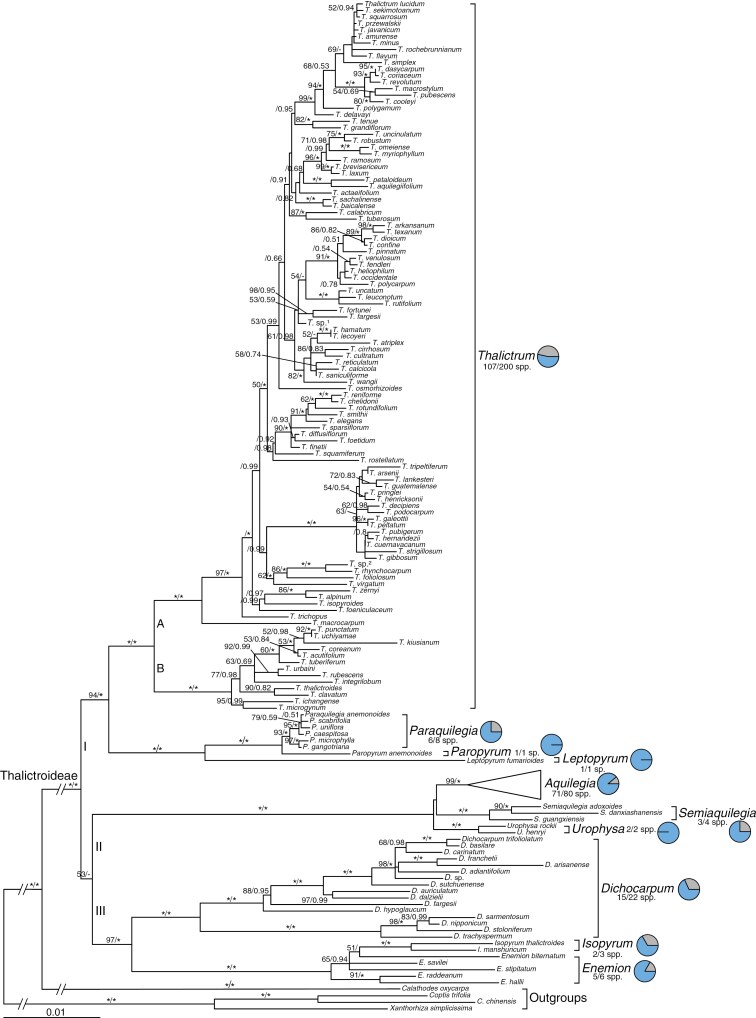
Maximum likelihood tree of Thalictroideae inferred from the combined plastid and ITS dataset. Bootstrap values (BS ≥ 50 %) and posterior probabilities (PP ≥ 0.5) are shown above the branches. Asterisks denote BS = 100 % or PP = 1.0. ‘–’ indicates the nodes not found in Bayesian tree. Pie charts on the right denote sampling fractions for each genus, with blue sectors indicating the proportions of sampled species.

The aligned plastid dataset comprised 6572 characters: *rbcL*, 1254 bp; *matK*, 1257 bp; *ndhA*, 1177 bp; *trnL*-*F*, 1161 bp; *atpB*-*rbcL*, 1162 bp; and *rpl32*-*trnL*, 561 bp. The aligned ITS sequences were 663 nucleotides in length. Our tree-based comparisons indicated that except for several nodes with weak support (PP < 0.95 or BS < 70 %), phylogenetic relationships recovered by the plastid dataset were highly congruent with those obtained from the ITS dataset (Supplementary Data Figs S1–S3). The combined plastid and ITS dataset consisted of 7235 characters. The BI and ML analyses generated highly congruent topologies, with strong support for most nodes (Fig. 1). Thalictroideae is strongly supported as monophyletic (BS = 100 %, PP = 1.0). Within Thalictroideae, three major clades (I, II and III) are recognized. Clade I contains *Leptopyrum*, *Paraquilegia*, *Paropyrum* and *Thalictrum* (BS = 94 %, PP = 1.0); clade II consists of *Aquilegia*, *Semiaquilegia* and *Urophysa* (BS = 100 %, PP = 1.0); and clade III includes *Dichocarpum*, *Enemion* and *Isopyrum* (BS = 97 %, PP = 1.0). *Thalictrum*, *Leptopyrum* and *Paropyrum* are successive sister taxa to *Paraquilegia*. *Semiaquilegia* is sister to *Aquilegia* with poor support. *Isopyrum* and *Enemion* form a clade (BS = 100 %, PP = 1.0). All genera for which at least two species were sampled are strongly supported as monophyletic except for *Isopyrum*. *Isopyrum* is deeply embedded within *Enemion* and is sister to *Enemion biternatum* with weak support. Within the most species-rich genus, *Thalictrum*, two major subclades (A and B) are recovered.

### Divergence times

Divergence time estimates for Thalictroideae are shown in Supplementary Data Fig. S4. The crown group age of Thalictroideae was estimated at 36.37 Mya (95 % HPD: 29.44–42.60 Mya). In Thalictroideae, three major clades diverged rapidly between 36 and 32 Mya. In clade I, *Thalictrum* split from other three genera at 26.84 Mya (95 % HPD: 19.83–33.22 Mya), *Leptopyrum* occurred at 15.36 Mya (95 % HPD: 9.50–22.19 Mya), and *Paropyrum* and *Paraquilegia* diverged at 4.33 Mya (9 5% HPD: 2.32–7.20 Mya). The crown group ages of *Paraquilegia* and *Thalictrum* were estimated at 2.33 Mya (95 % HPD: 1.09–3.89 Mya) and 20.44 Mya (95 % HPD: 14.97–26.51 Mya), respectively. The stem and crown group ages of *Dichocarpum* were estimated at 28.45 Mya (95 % HPD: 22.48–34.91 Mya) and 22.33 Mya (95 % HPD: 17.10–28.50 Mya), respectively. *Urophysa*, *Semiaquilegia* and *Aquilegia* diverged rapidly within a 1 Myr time window (~8–9 Mya). *Aquilegia* began to diversify at 2.93 Mya (95 % HPD: 1.49–4.84 Mya).

### Ancestral ranges and habitat types

**Fig. 2.**
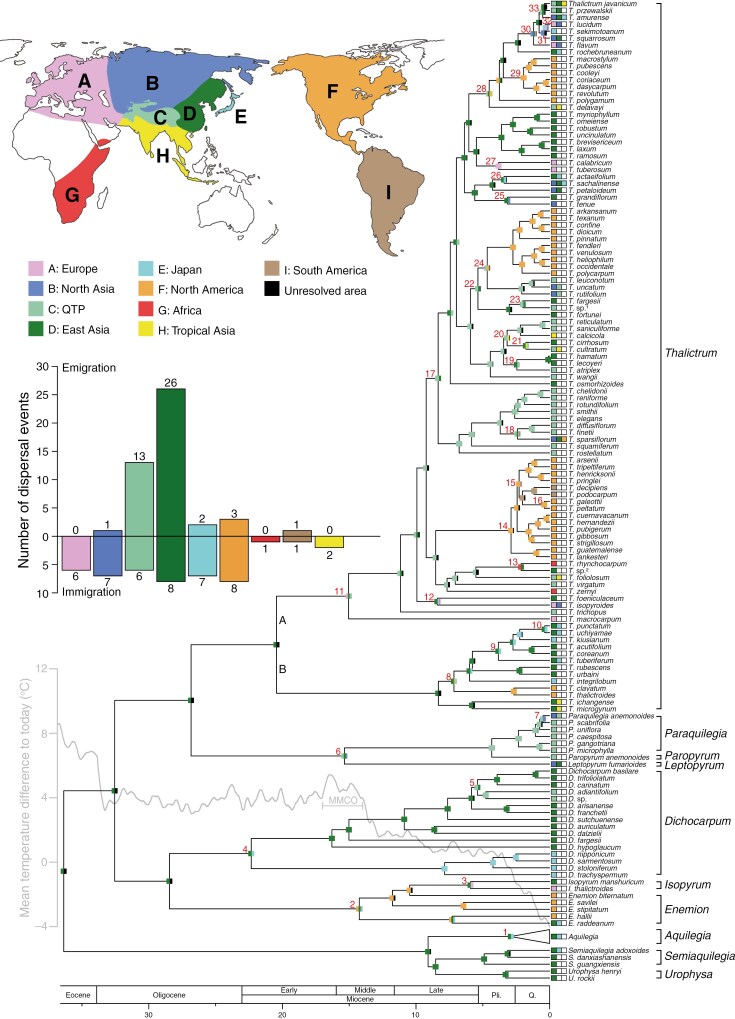
Ancestral range reconstruction on the Thalictroideae chronogram under the DEC+*j* model. Boxes on each node show cumulative probabilities for estimated ancestral ranges. Nodes with dispersal events are marked as 1–33, as referred to in Supplementary Data Table S8. The bar chart shows the numbers of emigration and immigration events of each area. The depiction of temperature changes is modified from Westerhold *et al.* (2020). Abbreviations: MMCO, the middle Miocene Climate Optimum; Pli., Pliocene; Q., Quaternary.

Ancestral range estimations under the DEC+*j* (Fig. 2) and DEC (Supplementary Data Fig. S5) models yielded highly congruent results. The DEC+*j* model was optimal for our data (Supplementary Data Table S5), hence the results from the DEC+*j* model are reported and used for discussion. The most recent common ancestor (MRCA) of Thalictroideae was likely to be present in East Asia. The distribution of the subfamily is inferred to be the result of ≥46 dispersal events, all of which happened from the early Miocene onwards (Fig. 2; Supplementary Data Table S9): six from East Asia to Japan (nodes 1, 2, 4, 8, 9 and 26), six from East Asia to the QTP (nodes 5, 6, 11, 23, 28 and 33), five from East Asia to North Asia (nodes 6, 12, 25, 30 and 31), four from East Asia to North America (nodes 2, 8, 28 and 29), four from East Asia to Europe (nodes 11, 12, 27 and 30), one from East Asia to Tropical Asia (node 28), six from the QTP to East Asia (nodes 13, 17, 18, 19, 21 and 22), two from the QTP to North Asia (nodes 7 and 18), three from the QTP to North America (nodes 14, 18 and 24), one from the QTP to Tropical Asia (node 20), one from the QTP to Africa (node 13), one from North Asia to Japan (node 32), one from North Asia to Europe (node 32), one from Japan to East Asia (node 10), one from North America to East Asia (node 3), one from North America to Europe (node 3), one from North America to South America (node 15) and one from South America to North America (node 16). Our ancestral habitat reconstruction shows that the MRCA of Thalictroideae was likely to be present in closed habitat, and independently colonized open habitat at least five times, of which four occurred in *Thalictrum* (Supplementary Data Fig. S6).

### Rates of diversification

**Fig. 3.**
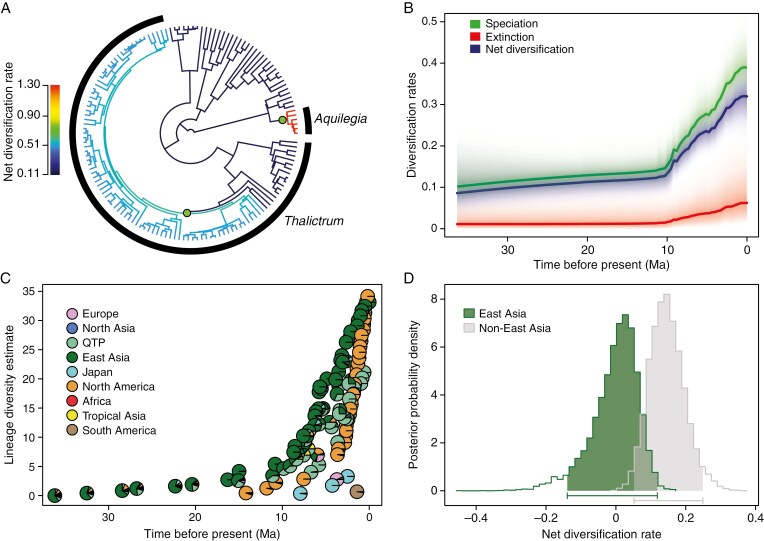
Diversification dynamics of Thalictroideae. (A) Phylorate plot with branches coloured by net diversification rate inferred from BAMM analysis. Green dots indicate the positions of the significant net diversification rate increases in the best shift configuration. (B) Rate-through-time plots for speciation, extinction and net diversification rates obtained from the BAMM analysis. Colour density shadings denote confidence in rate reconstructions. (C) Region-specific lineage diversity plots. (D) Posterior probability distributions of net diversification rates based on Bayesian GeoSSE analyses. Shaded areas and horizontal bars indicate 95 % confidence intervals.

The BAMM analyses detected a non-constant diversification process across Thalictroideae with the background speciation rate of 0.24 lineages Myr^−1^. The best shift configuration shows that net diversification rates of the subfamily experienced two rapid accelerations (Fig. 3A): one within *Thalictrum* (~10 Mya; 0.40 lineages Myr^−1^) and the other at the crown of *Aquilegia* (~3 Mya; 0.74 lineages Myr^−1^). Rates through time curves indicate a sharp rise of diversification rates at ~10 Mya (Fig. 3B). Region-specific lineage diversity plots show that rapid lineage accumulation in both East Asia and QTP occurred at ~10 Mya and in North America at ~3 Mya (Fig. 3C). The GeoSSE analysis shows that net diversification rate of East Asia was lower than that of non-East Asia (Fig. 3D).

## DISCUSSION

### Phylogeny of Thalictroideae

This study provides a species-level phylogenetic framework for Thalictroideae with the most comprehensive taxon sampling to date (Fig. 1), which is a foundation for the future taxonomic revision for this subfamily. Thalictroideae is strongly supported as monophyletic, which is consistent with previous molecular studies (e.g. Wang and Chen, 2007; Cossard *et al.*, 2016; Wang *et al.*, 2016). Within Thalictroideae, three major clades (clade I, II and III) were recovered, in agreement with the results of Wang and Chen (2007) but with higher support. Our results show that *Dichocarpum* is sister to the *Enemion*–*Isopyrum* clade (BS = 100 %, PP = 1.0), which is congruent with the results of Wang and Chen (2007). Zhai *et al.* (2019) recovered *Dichocarpum* to be the sister group of the clade containing *Aquilegia*, *Enemion*, *Isopyrum*, *Semiaquilegia* and *Urophysa* (MP BS = 57 %, ML BS = 100 %, PP = 1.0), but they sampled only one species of *Dichocarpum*. Based on molecular and morphological data, Wang and Chen (2007) separated *Isopyrum anemonoides* from *Isopyrum* and recognized the monotypic *Paropyrum*, in agreement with our phylogenetic analyses. Most genera of Thalictroideae with more than one species are recognized as monophyletic except for *Isopyrum*. Wang and Chen (2007) sampled one *Enemion* species and identified the sister relationship between *Isopyrum* (excluding *Isopyrum anemonoides*) and *Enemion*. In this study, we sampled five of six species of *Enemion* and found that *Isopyrum* was nested in *Enemion* and sister to *E. biternatum* with weak support. Further study with more sampling (including taxon density and genes) is needed to determine the position of *Isopyrum* in *Enemion*.

### Biogeography of Thalictroideae

Our time estimates under the optimal relaxed clock model and tree prior suggest a crown group of 36.37 Mya (95 % HPD: 29.44–42.60 Mya) for Thalictroideae, which is in accordance with the estimates of Wang *et al.* (2016; 33.81 Mya, 95 % HPD: 27.62–41.24 Mya) and Zhai *et al.* (2019; 38.6 Mya), but is much older than that of Bastida *et al.* (2010; 27.57 Mya, 95 % HPD: 26.59–28.56 Mya). However, Bastida *et al.* (2010) sampled only 46 Thalictroideae species and used three DNA regions. Sufficient taxon density, not simply numerous base pairs, is crucial for reliable estimation of ages (Jian *et al.*, 2008; Xiang *et al.*, 2018). *Dichocarpum* started to diversify at 22.33 Mya (95 % HPD: 17.10–28.50 Mya), which coincides with the estimate of Xiang *et al.* (2017; 21.86 Mya, 95 % HPD: 16.36–27.32 Mya). The crown group age of *Thalictrum* is estimated to be 20.44 Mya (95 % HPD: 14.97–26.51 Mya), which is highly congruent with the result of Soza *et al.* (2013; 18.4 Mya, 95 % HPD: 14.00–22.80 Mya). Therefore, our divergence time estimates for Thalictroideae should be reliable.

Our molecular dating and ancestral range estimation analyses indicate that the MRCA of Thalictroideae occurred in East Asia in the late Eocene (~36 Mya), but did not disperse into other Northern Hemisphere regions until the early Miocene (Fig. 2; Supplementary Data Table S9). At least 46 dispersal events need to be invoked to explain the current distribution of Thalictroideae (Fig. 2), of which 45 occurred after the middle Miocene Climatic Optimum (~17–14 Mya; Westerhold *et al.*, 2020). Among the 46 dispersal events inferred in Thalictroideae, 34 are associated with East Asia, including 26 out-of-East Asian dispersal events and eight migrations back to East Asia (Fig. 2). These results suggest that Thalictroideae exemplifies the ‘out of and in East Asia’ biogeographical hypothesis. Previous studies have also illustrated that the dispersals between East Asia and Europe and between East Asia and North America were bidirectional (Yoo *et al.*, 2018; Zhou *et al.*, 2020). Thus, East Asia functions not merely as a source, but also as a sink of plant diversity of the Northern Hemisphere.

Five major floristic exchange routes were recognized to connect East Asia and other regions in the Northern Hemisphere. The first route involved five migrations to North Asia (nodes 6, 12, 25, 30 and 31) with subsequently westward dispersal into Europe (nodes 12, 27 and 30). A similar dispersal scenario is also recognized in *Gentiana* (Gentianaceae; Favre *et al.*, 2016). The second dispersal route connected East Asia and North America, with four migrations from East Asia to North America (nodes 2, 8, 28 and 29) and one migration from North America to East Asia (node 3). After the middle Miocene, the North Atlantic land bridge had been sundered, but the Bering land bridge was still available (Tiffney, 1985*a*, *b*; Tiffney and Manchester, 2001; Wen *et al.*, 2010). Therefore, it is more likely that Thalictroideae first arrived in North Asia and moved onwards to North America through the Bering land bridge. The third dispersal passage promoted bidirectional exchanges between East Asia and the QTP, including six migrations from East Asia to the QTP (nodes 5, 6, 11, 23, 28 and 33) and six reverse (nodes 13, 17, 18, 19, 21 and 22). Interestingly, the subclade A of *Thalictrum* probably leveraged the QTP as the transit point to reach Europe (node 11), which has also been proposed for *Quercus* section *Ilex* (Jiang *et al.*, 2019). The fourth route allowed population exchanges of Thalictroideae between East Asia and Japan, including six dispersals from East Asia to Japan (nodes 1, 2, 4, 8, 9 and 26) and one back to East Asia (node 10). Along with the drop of sea level (Miller *et al.*, 2020), the seafloor exposure of the East China Sea and the Sea of Japan might provide plant dispersal corridors between East Asia and the Japanese Islands (Millien-Parra and Jaeger, 1999; Qiu *et al.*, 2009). Similar dispersal scenarios are also recognized in other plant taxa, such as *Pseudotsuga* (Pinaceae; Wei *et al*., 2010), *Euptelea* (Eupteleaceae; Cao *et al.*, 2016), *Coptis* (Ranunculaceae; Xiang *et al.*, 2018) and *Actaea* (Ranunculaceae; Ling *et al.*, 2023). The fifth route led to the southward range expansion of Thalictroideae to Tropical Asia in the Pliocene (4.52 Mya, 95 % HPD: 3.15–5.97 Mya; node 28). Favre *et al.* (2016) reported multiple migrations from East Asia to Tropical Asia in *Gentiana* (Gentianaceae) during the same period.

Our results also show that the QTP frequently had floristic exchanges with other geographical areas (Fig. 2). Apart from the colonization of adjacent areas, such as North Asia (node 7) and Tropical Asia (node 20), there were four intercontinental out-of-QTP events in Thalictroideae. Three from the QTP to North America occurred in the Pliocene to Pleistocene (nodes 14, 18 and 24), which was possibly aided by the transitional colonization in North Asia. As a result of the fluctuation of sea level, the Bering land bridge was available intermittently until the Quaternary glaciations (Hopkins, 1972; Brikiatis, 2014; Miller *et al.*, 2020). Another intercontinental dispersal was inferred from the QTP to Africa at ~0.79–3.69 Ma (node 13), which coincides temporally with the establishment of a new floristic exchange route between southwestern Asia and eastern Africa through the Arabian Peninsula and the Levant region in the Pliocene (Fernandes *et al.*, 2006). Similar exchanges between the QTP and Africa via this passageway have also been found in *Lepidium* of Brassicaceae (1.8–3.6 Mya; Mummenhoff *et al.*, 2001) and in Loliinae (2.5–3.6 Mya; Inda *et al.*, 2008) and Bambusoideae (3.4–5.1 Mya; Zhang *et al.*, 2016) of Poaceae.

In addition, we found that *Thalictrum* dispersed from North America to South America (node 15) and back to North America (node 16) during the Quaternary. Along with the complete closure of the Isthmus of Panama in the early Pliocene (~3–4 Mya; Weir *et al.*, 2009; Leigh *et al.*, 2014; Jaramillo *et al.*, 2017), the terrestrial connection of North and South America was fully established and thus might trigger the exchanges of Thalictroideae populations between two continents. The Isthmus of Panama has played a crucial role in plant exchanges for other temperate and tropical taxa, such as *Philodendron* (Araceae; Canal *et al.*, 2019) and *Clowesia* (Orchidaceae; Tamayo-Cen *et al.*, 2022).

### Diversification of Thalictroideae

Our diversification analyses indicate that the MRCA of Thalictroideae occupied East Asian forests in the late Eocene (~36 Mya) and rapidly diverged *in situ* into three major clades in a period of ~4 Myr, perhaps in as little as 1 Myr (Fig. 2; Supplementary Data Fig. S6). During the late Eocene–early Oligocene, global temperature dropped sharply (Westerhold *et al.*, 2020), and aridification in Central Asia began to occur (Bosboom *et al.*, 2014). These events probably caused the southward retreat of evergreen forests and facilitated the development of temperate deciduous forests in Asia (Ling *et al.*, 2023), which might create many new ecological niches for the early diversification of Thalictroideae. A few plant lineages in East Asian deciduous forests, such as *Fagus* (Fagaceae; Hai *et al.*, 2022) and *Actaea* (Ranunculaceae; Ling *et al.*, 2023), also diversified rapidly during the same period.

The region-specific lineage diversity estimation shows that East Asian Thalictroideae lineages underwent a steady accumulation from 36 to 10 Mya, corresponding to the ‘museum’ model. From the late Eocene onwards, the climatic deterioration happened multiple times, particularly after the middle Miocene Climatic Optimum (Westerhold *et al.*, 2020). Nevertheless, ecological niche modelling analyses of 442 relict species in East Asia support the existence of long-term climatically stable refugia (Tang *et al.*, 2018), which might have protected plants from extinction under dramatic climate changes and facilitated the steady accumulation of East Asian Thalictroideae species from 36 to 10 Mya. The BAMM analyses indicate a dramatic increase of diversification rates in Thalictroideae at ~10 Mya (Fig. 3B), temporally consistent with the rapid accumulation of East Asian lineages (Fig. 3C). The establishment of a ‘super monsoon’ in East Asia during the late Miocene (~12–4 Mya; Farnsworth *et al.*, 2019) brought high precipitation and might have contributed to accelerated diversification of Thalictroideae. In addition, the GeoSSE analysis indicates that the net diversification rate of East Asia was lower than that of non-East Asia (Fig. 3D). These findings suggest that rich species diversity of Thalictroideae in East Asia might benefit not only from the gradual accumulation of lineages during the late Eocene-middle Miocene but also from the rapid radiation from the late Miocene onwards. Thus, we propose that East Asia might have served as both an evolutionary museum and a cradle for the diversity of Thalictroideae and probably for other herbaceous lineages.

Our diversification analyses also show that the QTP lineages diversified rapidly at ~10 Mya (Fig. 3C), which might be ascribed mainly to a simultaneous shift of net diversification rate detected in subclade A of *Thalictrum* (0.40 vs. 0.24 lineages Myr^−1^; Fig. 3A). This timing coincides with the broad-scale uplift of the QTP and adjacent regions in the late Miocene (Zeitler, 1985; Liu *et al.*, 1996; Wang *et al.*, 2014*a*; Miao *et al.*, 2022; Yan *et al.*, 2024), which might result in complex topography and the occurrence of diverse habitats that promoted the diversification of Thalictroideae in the QTP.

## Conclusion

Our study presents a comprehensive phylogeny for Thalictroideae with 217 taxa based on seven DNA regions from plastid and nuclear genomes. Our analyses indicate that Thalictroideae originated in the late Eocene of East Asia and that 46 dispersal events from the early Miocene onwards shaped the current distribution and endemism of this subfamily. We detected 26 out-of-East Asian dispersal events and eight migrations back to East Asia, suggesting that East Asia has acted as both a source and a sink of plant diversity of the Northern Hemisphere. Importantly, our diversification analyses show that East Asian Thalictroideae lineages underwent a steady accumulation from 36 to 10 Mya and experienced a rapid accumulation at ~10 Mya, implying that East Asia might have functioned as both an evolutionary museum and a cradle for the diversity of Thalictroideae and probably for other herbaceous lineages. Above all, this study highlights a complicated role of East Asia in shaping the biogeographical patterns of the Northern Hemisphere and provides new insights into the historical assembly of East Asian biodiversity.

## SUPPLEMENTARY DATA

Supplementary data are available at *Annals of Botany* online and consist of the following.

Figure S1: maximum likelihood tree of Thalictroideae inferred from the plastid dataset. Figure S2: maximum likelihood tree of Thalictroideae inferred from the ITS dataset. Figure S3: comparison of topologies for Thalictroideae obtained from the plastid (A) and ITS (B) datasets. Figure S4: chronogram Thalictroideae based on the combined plastid and ITS dataset using BEAST. Figure S5: ancestral range reconstruction for Thalictroideae under the DEC model. Figure S6: ancestral habitat reconstruction for Thalictroideae. Table S1: taxa, vouchers, localities and GenBank accession numbers for the sequences used in this study. Table S2: primers used for amplification and sequencing in this study. Table S3: comparison of four clock models in BEAST analyses via Bayes factors. Table S4: comparison of two tree prior processes in BEAST analyses via Bayes factors. Table S5: comparison of the fit of different models of biogeographical range evolution and model-specific estimates for the different parameters. Table S6: dispersal multiplier matrix used in ancestral range reconstruction. Table S7: species, designated terminal clade and proportion of the number of extant species sampled per terminal in the BAMM analysis. Table S8: comparison of the fit of different models of GeoSSE analysis. Table S9: estimated ages for the nodes with dispersal events.

mcae148_suppl_Supplementary_Material
