# Supplementary material for: Out of and in East Asia: phylogeny, biogeography and diversification of Thalictroideae (Ranunculaceae) in the Northern Hemisphere
Source: Ann Bot. 2024 Aug 28;134(7):1251–62. doi: 10.1093/aob/mcae148 (PMC11688531; doi:10.1093/aob/mcae148)
Supplement: mcae148_suppl_Supplementary_Material [file mcae148_suppl_supplementary_material.pdf]

SUPPLEMENTARY DATA

FIGURE S1. ML tree of Thalictroideae inferred from the plastid dataset.

FIGURE S2. ML tree of Thalictroideae inferred from the ITS dataset.

FIGURE S3. Comparison of topologies for Thalictroideae obtained from the plastid (A) and ITS (B) datasets.

FIGURE S4. Chronogram Thalictroideae based on the combined plastid and ITS dataset using BEAST.

FIGURE S5. Ancestral range reconstruction for Thalictroideae under the DEC model.

FIGURE S6. Ancestral habitat reconstruction for Thalictroideae.

TABLE S1. Taxa, vouchers, localities and GenBank accession numbers for the sequences used in this study.

TABLE S2. Primers used for amplification and sequencing in this study.

TABLE S3. Comparison of four clock models in BEAST analyses via Bayes factors.

TABLE S4. Comparison of two tree prior processes in BEAST analyses via Bayes factors.

TABLE S5. Comparison of the fit of different models of biogeographical range evolution and model-specific estimates for the different parameters.

TABLE S6. Dispersal multiplier matrix used in ancestral range reconstruction.

TABLE S7. Species, designated terminal clade, and proportion of the number of extant species sampled per terminal in the BAMM analysis.

TABLE S8. Comparison of the fit of different models of GeoSSE analysis.

TABLE S9. Estimated ages for the nodes with dispersal events.

Legends for Figures S1–S3

FIGURE S1. ML tree of Thalictroideae inferred from the plastid dataset. Bootstrap values (BS ≥ 50 %) and posterior probabilities (PP ≥ 0.5) are shown above the branches. Asterisks denote BS = 100% or PP = 1.0. "–" indicates the nodes not found in Bayesian tree.

FIGURE S2. ML tree of Thalictroideae inferred from the ITS dataset. Bootstrap values (BS ≥ 50 %) and posterior probabilities (PP ≥ 0.5) are shown above the branches. Asterisks denote BS = 100% or PP = 1.0.

FIGURE S3. Comparison of topologies for Thalictroideae obtained from the plastid (A) and ITS (B) datasets. Numbers above branches are bootstrap values (BS) and posterior probabilities (PP). Following the suggestion of Wang *et al*. (2014), only nodes with BS ≥ 70% and PP ≥ 0.95 were indicated. Asterisks denote BS = 100% or PP = 1.0.

FIGURE S4. Chronogram Thalictroideae based on the combined plastid and ITS dataset using BEAST. Estimated ages are shown above branches with 95% highest posterior density intervals (grey bars). Nodes of interest were marked as 1–33. Red dots show three secondary calibration points (see details in the Text). Pli., Pliocene; Q., Quaternary.

FIGURE S5. Ancestral range reconstruction for Thalictroideae under the DEC model. The cumulative probabilities for estimated ancestral ranges were obtained in BioGeoBEARS, implemented in RASP (see details in Text). Numbers in red near branches indicate the node number, as referred to Fig. 2. Pli., Pliocene; Q., Quaternary.

FIGURE S6. Ancestral habitat reconstruction for Thalictroideae. Pie charts at each node show the relative probabilities of alternative ancestral states. Numbers in red near branches indicate the node number, as referred to Fig. 2. Pli., Pliocene; Q., Quaternary.

TABLE S1. *Taxa, vouchers, localities and GenBank accession numbers for the sequences used in this study*

Taxon	Voucher	Locality	*rbcL*	*matK*	*ndhA*	*trnL-F*	*atpB-rbcL*	*rpl32-trnL*	ITS
Aquilegia alpina L.				KC290052	KC288720			KC290230	EU729330
Aquilegia amurensis Kom.	Zhukova *s.n.* (MHA)	Russia	PQ143361	PQ143446	PQ143606	PQ143520	PQ143783	PQ143699	PQ144357
Aquilegia apuana (Marchetti) E.Nardi	Marchetti D. & Trombetti G. *s.n.* (FI)	Italy			PQ143606	PQ143521	PQ143784	PQ143700	PQ144358
Aquilegia aradanica Shaulo & Erst	Shaulo D. & Erst A.S. *s.n.* (NS)	Russia	PQ143362	PQ143447	PQ143608	PQ143522	PQ143785	PQ143701	PQ144359
Aquilegia atrata W.D.J.Koch			FR865154	KC290057	KC288725			KC290235	EU729331
Aquilegia aurea Janka	Bozilova E. 19751807	Bulgaria	PQ143363	PQ143448	PQ143609	PQ143523	PQ143786	PQ143702	PQ144360
Aquilegia barbaricina Arrigoni & E.Nardi				KC290060	KC288728			KC290238	EU729332
Aquilegia barnebyi Munz	Goodrich S. 00661843 (NY)	U.S.A.	NC_053820	PQ143449	PQ143610	PQ143524	PQ143787	PQ143703	PQ144361
Aquilegia barykinae Erst, Karakulov & Luferov	Karakulov A. 20102306 (NS)	Russia	PQ143364	PQ143450	PQ143611	PQ143525	PQ143788	PQ143704	PQ144362
Aquilegia bernardii Gren. & Godr.				KC290061	KC288729			KC290239	EU729333
Aquilegia borodinii Schischk.	Artemov I. 20150407 (NS)	Russia	PQ143365	PQ143451	PQ143612	PQ143526	PQ143789	PQ143705	PQ144363
Aquilegia brevistyla Hook.			DQ099444	KC290072	KC288740			KC290250	
Aquilegia buergeriana Siebold & Zucc.			LC617122	LC617155	KC288741			KC290251	JX233769
Aquilegia caerulea E.James	Chen Z.D. & Kong H.Z. CL104 (PE)	U.S.A.	PQ143366	PQ143452	PQ143613	PQ143527	PQ143790	PQ143706	PQ144364
Aquilegia canadensis L.	Liu Y. *s.n.* (PE)	U.S.A.	PQ143367	PQ143453	PQ143614	PQ143528	PQ143791		PQ144365
Aquilegia champagnatii Moraldo, Nardi & la Valva	Fior S. & Lega M. 010 (ROV)	Italy	PQ143368	PQ143454	PQ143615	PQ143529	PQ143792	PQ143707	PQ144366
Aquilegia chrysantha A.Gray			JN796929	KC290076	KC288744	MT919113	MT919113	KC290254	Hoges 1994
Aquilegia colchica Kem.-Nath.	Vassiljeva L. *s.n.* (LE)	Georgia			PQ143616	PQ143530	PQ143793	PQ143708	PQ144367
Aquilegia confusa Rota	Prosser F. *s.n.* (ROV)	Italy	PQ143369	PQ143455	PQ143617	PQ143531	PQ143794	PQ143709	PQ144368
Aquilegia cremnophila Bacch., Brullo, Congiu, Fenu, J.L.Garrido & Mattana	Bacchetta G. et al. (FI)	Italy	PQ143370	PQ143456	PQ143618	PQ143532	PQ143795	PQ143710	PQ144369
Aquilegia daingolica Erst & Shaulo	Kamelin R.V. et al. 1665 (NS)	Mongolia	PQ143371		PQ143619	PQ143533	PQ143796	PQ143711	
Aquilegia ecalcarata Maxim.	Wang W. 117 (PE)	China	PQ143372	PQ143457	PQ143620	PQ143534	PQ143797	PQ143712	PQ144370
Aquilegia einseleana F.W.Schultz				KC290065	KC288733			KC290243	EU729329
Aquilegia elegantula Greene				KC290081	KC288749			KC290259	EU747251
Aquilegia flabellata Siebold & Zucc.	Konovalova T. *s.n.* (MHA)	Russia	PQ143373		PQ143621	PQ143535	PQ143798	PQ143713	PQ144371

Aquilegia flavescens S.Watson				KC290084	KC288752			KC290262	Hoges 1994
Aquilegia formosa Fisch. ex DC.			MT919116	KC290085	KC288753	KP900425	MT919116	KC290263	KC417070
Aquilegia fragrans Benth.	Pakistan Expedition FPH-0471(PE)	Pakistan	PQ143374	PQ143458	PQ143622	PQ143536	PQ143799	PQ143714	PQ144372
Aquilegia ganboldii Kamelin & Gubanov	Ganbold E. 1310 (NS)	Mongolia	PQ143375		PQ143623	PQ143537	PQ143800	PQ143715	PQ144373
Aquilegia gegica Jabr.-Kolak.	Vassiljeva L. *s.n.* (LE)	Russia	PQ143376	PQ143459	PQ143624	PQ143538	PQ143801	PQ143716	PQ144374
Aquilegia glandulosa Fisch. ex Link.				KC290087	KC288755			KC290265	EU747253
Aquilegia hebeica[1] Erst	Peng H.W. & Zhang J. *s.n.* (PE)	China	PQ143377	PQ143460	PQ143625	PQ143539	PQ143802		PQ144375
Aquilegia hebeica[2] Erst	Erst A.S. *s.n.* (NS)	China	PQ143378	PQ143461	PQ143626	PQ143540	PQ143809		PQ144431
Aquilegia incurvata P.K.Hsiao	Zhang Q. et al. XYB0607-190 (PE)	China	PQ143379	PQ143462	PQ143627	PQ143541	PQ143803	PQ143717	PQ144376
Aquilegia japonica Nakai & H.Hara	Li L.F. 201106 (PE)	China	PQ143380	PQ143463	KC288751	PQ143542	MT919110	PQ143718	
Aquilegia jonesii Parry				KC290089	KC288757			KC290267	Ro 1996
Aquilegia jucunda Fisch. & Avé-Lall.	Zibzeev E. & Basargin E. *s.n.* (NS)	Kazakhstan	PQ143381	PQ143464	PQ143628	PQ143543	PQ143804	PQ143719	PQ144377
Aquilegia kamelinii Erst, Shaulo & Shmakov	Boiko & Starchenko *s.n.* (MHA)	Russia	PQ143382	PQ143465	PQ143629	PQ143544	PQ143805	PQ143720	PQ144378
Aquilegia karatavica Mikeschin	Kamelin R. 1548 (LE)	Kazakhstan	PQ143383	PQ143466	PQ143630	PQ143545	PQ143806		PQ144379
Aquilegia kubanica I.M.Vassiljeva	Zernov A. et al. 7627a (NS)[1]; Gogina 433 (MHA)[2]	Russia[1]; Russia[2]	PQ143384[1]	PQ143467[1]	PQ143631[1]	PQ143546[1]	PQ143807[1]	PQ143721[2]	PQ144380[2]
Aquilegia lactiflora Kar. & Kir.	Lazkov G. & Kentenbaeva N. *s.n.*	Kyrgyzstan			PQ143632	PQ143547	PQ143808	PQ143722	PQ144381
Aquilegia longissima A.Gray ex S.Watson			OL537535	KC290094	KC288762			KC290272	OL537301
Aquilegia lucensis E.Nardi	Foggi B. *s.n.* (FI)	Italy	PQ143385	PQ143468	PQ143633	PQ143548	PQ143810	PQ143723	PQ144432
Aquilegia nevadensis Boiss. & Reut.	Valdes B. et al. It2392/88 (FI)	Spain	PQ143386		PQ143634	PQ143549	PQ143811	PQ143724	PQ144382
Aquilegia nigricans Baumg.				KC290097	KC288766			KC290275	EU747255
Aquilegia nivalis (Falc. ex Brühl) J.R.Drumm. & Hutch.	Pakistan Expedition FPH-0743 (PE)	Pakistan	PQ143387	PQ143469	PQ143635	PQ143550	PQ143812	PQ143725	PQ144383
Aquilegia nugorensis Arrigoni & E.Nardi				KC290099	KC288767			KC290277	EU747256
Aquilegia nuragica Arrigoni & E.Nardi	Arrigoni P.V. & Nardi E. 10 (FI)	Italy			KC288768	PQ143551	PQ143813	PQ143726	
Aquilegia olympica Boiss.				KC290101	KC288769			KC290279	EU676169
Aquilegia ophiolithica Barberis & E.Nardi	Marchetti D. 20111406 (FI)	Italy	PQ143388	PQ143470	PQ143636	PQ143552	PQ143814	PQ143727	PQ144384
Aquilegia ottonis Orph. ex Boiss.				KC290075	KC288743			KC290253	EU747257
Aquilegia oxysepala Trautv. & C.A.Mey.	Chen Z.D. *s.n.* (PE)	China	EF437140	EF437128	PQ143637	EF437097	PQ143815	JX258540	EF437114

Aquilegia parviflora Ledeb.	Li L.F. 201104 (PE)	China	PQ143389	PQ143471	PQ143638	PQ143553	PQ143816	KC290286	PQ144385
Aquilegia pubescens Coville				KC290106	KC288774			KC290283	Ro 1996
Aquilegia pubiflora Wall. ex Royle	Pakistan Expedition FPH-1251 (PE)	Pakistan	PQ143390	PQ143472	PQ143639	PQ143554	PQ143817	PQ143728	PQ144386
Aquilegia pyrenaica DC.				KC290108	KC288776			KC290285	EU747258
Aquilegia rockii Munz			MK573514	MK573514	MK573514	MK573514	MK573514	MK573514	MF785638
Aquilegia saximontana Rydb.	Roh M.S. 20041807 (NS)	U.S.A.		PQ143473	PQ143640	PQ143555	PQ143818	PQ143729	PQ144387
Aquilegia scopulorum Tidestr.				KC290111	KC288779			KC290288	EU747242
Aquilegia shockleyi Eastw.	Morefield J.D. & McCarty 3782 (NS)	U.S.A.	PQ143391	PQ143474	PQ143641	PQ143556	PQ143819	PQ143730	PQ144388
Aquilegia sibirica Lam.			MT919115	KC290112	KC288780	MT919115	MT919115	KC290289	EU747259
Aquilegia sicula (Strobl) E.Nardi	Bavazzano R. 19722205 (FI)	Italy		PQ143475	PQ143642	PQ143557	PQ143820		PQ144389
Aquilegia skinneri Hook.				KC290113	KC288781			KC290290	Hoges 1994
Aquilegia thalictrifolia Rydb.				KC290115	KC288791			KC290300	EU747243
Aquilegia turczaninowii Kamelin & Gubanov	Kondrat'eva E. *s.n.* (NS)	Russia	PQ143392		PQ143643	PQ143558	PQ143821	PQ143731	PQ144390
Aquilegia viridiflora Pall.	Bashan Expedition 5032 (PE)	China	PQ143393	PQ143476	PQ143644	PQ143559	PQ143822	PQ143732	PQ144391
Aquilegia viscosa Gouan				KC290127	KC288795			KC290304	EU747246
Aquilegia vitalii Gamajun.	Rusanovich I.T. & Kramarenko L. *s.n.* (MHA)	Kazakhstan	PQ143394	PQ143477	PQ143645	PQ143560	PQ143823	PQ143733	PQ144392
Aquilegia vulgaris L.			EU053905	KC290133	KC288797	MT919112	MT919112	KC290310	EU747245
Aquilegia wittmanniana Steven ex Fisch., C.A.Mey. & Avé-Lall.	Patarana M. *s.n.* (LE)	Georgia	PQ143395	PQ143478	PQ143646	PQ143561	PQ143824	PQ143734	PQ144393
Aquilegia xinjiangensis Erst	Erst A.S. *s.n.* (NS)	China	PQ143396	PQ143479	PQ143698	PQ143562	PQ143825	PQ143735	PQ144394
Aquilegia yabeana Kitag.	Wang W. SX026 (PE)	China	PQ143397	PQ143480	PQ143647	PQ143563	PQ143826	PQ143736	PQ144395
Dichocarpum adiantifolium (Hook.f. & Thomson) W.T.Wang & P.K.Hsiao						KY235746			KY235684
Dichocarpum arisanense (Hayata) W.T.Wang & P.K.Hsiao	Chen Z.D. et al. 20110365 (PE)	China	PQ143398	KY235700		KY235744	PQ143828	PQ143737	KY235682
Dichocarpum auriculatum (Franch.) W.T.Wang & P.K.Hsiao	Zhu D.H. et al. 2755 (PE)	China		KY235703		KY235748	PQ143829		KY235686
Dichocarpum basilare W.T.Wang & P.K.Hsiao				JN605364					HQ844055
Dichocarpum carinatum D.Z.Fu	Fu D.Z. 84332 (PE)	China	PQ143399	KY235704		KY235749	PQ143830		KY235688

Dichocarpum dalzielii (J.R.Drumm. & Hutch.) W.T.Wang & P.K.Hsiao	Wang W. 111 (PE)	China	EF437141	EF437130	PQ143649	EF437098	PQ143831	PQ143738	EF437115
Dichocarpum fargesii (Franch.) W.T.Wang & P.K.Hsiao	Anonymous QL-152 (PE)	China	KU662954	JN605366	PQ143650	KY235751	PQ143832	PQ143739	KY235689
Dichocarpum franchetii (Finet & Gagnep.) W.T.Wang & P.K.Hsiao	Sanxia Expedition 732 (PE)	China	PQ143400	KY235708	PQ143651	KY235753	PQ143833	PQ143741	KY235691
Dichocarpum hypoglaucum W.T.Wang & P.K.Hsiao	Shui Y.M. et al. 20300 (PE)	China		KY235710	PQ143652	KY235755	PQ143834	PQ143740	KY235693
Dichocarpum nipponicum (Franch.) W.T.Wang & P.K.Hsiao	Kosuge 556 (PE)	Japan	PQ143401	KY235711		KY235756	PQ143835		KY235694
Dichocarpum sarmentosum (Ohwi) Murata	Nagamsn H. 4507 (HAST)	Japan	PQ143402	KY235712	PQ143653	KY235757	PQ143836		KY235695
Dichocarpum sp.	Liu E.D. 6396 (KUN)	China	PQ143403	KY235702		KY235747	PQ143837		KY235685
Dichocarpum stoloniferum (Maxim.) W.T.Wang & P.K.Hsiao		Japan		KY235713		KY235759			KY235697
Dichocarpum sutchuenense (Franch.) W.T.Wang & P.K.Hsiao	Wang W. 69 (PE)	China	PQ143404	KY235714	PQ143654	KY235760	PQ143838		EF437116
Dichocarpum trachyspermum (Maxim.) W.T.Wang & P.K.Hsiao	Murata J. & Chen T.T. 9697 (KUN)		PQ143405	KY235715		KY235761			KY235698
Dichocarpum trifoliolatum W.T.Wang & P.K.Hsiao				JN605369					HQ844062
Enemion biternatum Raf.	Phillippe L.R. 40579 (PE)	U.S.A.	PQ143406	PQ143481	PQ143655		PQ143839	PQ143782	
Enemion hallii (A.Gray) J.R.Drumm. & Hutch.	Hall 10 (P)	U.S.A.	PQ143407	PQ143482	PQ143656	PQ143564	PQ143840	PQ143742	PQ144396
Enemion raddeanum Regel	Chen Z.D. & Xu K.X. 2090 (PE)	China	AY954494	EF437131	PQ143657	EF437100	PQ143841	PQ143743	EF437117
Enemion savilei (Calder & Roy L.Taylor) Keener			MG248207						MG236427
Enemion stipitatum (A.Gray) J.R.Drumm. & Hutch.			MF963062	MF963439					MF963803
Isopyrum manshuricum (Kom.) Kom. ex W.T.Wang & P.K.Hsiao	Wang W. LN004 (PE)	China	EF437143	EF437133	PQ143658	EF437102	PQ143842	PQ143744	EF437119
Isopyrum thalictroides L.	Anonymous *s.n.* (PE)	Poland	EF437144	PQ143483		PQ143565	PQ143843	PQ143745	PQ144397
Leptopyrum fumarioides (L.) Rchb.			EF437145	KY235716	NC_041542	KY235762	NC_041542		KY235699
Paraquilegia anemonoides (Willd.) Ulbr.	Seregin A. 20162907 (MW)	Kirgizia	PQ143408	PQ143484	PQ143659	PQ143566	PQ143844		PQ144398
Paraquilegia caespitosa (Boiss. & Hohen.) J.R.Drumm. & Hutch.	Yi L. *s.n.* (PE)	China	PQ143409	PQ143485	PQ143660	PQ143567	PQ143845		PQ144399
Paraquilegia gangotriana Pusalkar & D.K.Singh	Anonymous *s.n.* (LE)	China	PQ143410	PQ143486	PQ143661	PQ143568	PQ143846		PQ144400

Paraquilegia microphylla (Royle) J.R.Drumm. & Hutch.	Li C.Y. 001 (PE)	China	EF437146	EF437136	PQ143662	EF437105	PQ143847		EF437122
Paraquilegia scabrifolia Pachom.	Puchkova E.G. 19621508 (NS)	Kyrgyzstan	PQ143411	PQ143487	PQ143663	PQ143569	PQ143848		PQ144401
Paraquilegia uniflora (Aitch. & Hemsl.) J.R.Drumm. & Hutch.	Rubzov V. 19330208 (LE)	Kazakhstan	PQ143412	PQ143488	PQ143664	PQ143570	PQ143849		
Paropyrum anemonoides (Kar. & Kir.) Ulbr.	Wang Z.T. et al. 146 (PE)	China	PQ143413	PQ143489	PQ143665	PQ143571	PQ143850		PQ144402
Semiaquilegia adoxoides (DC.) Makino	Shao Q. 02 (PE)	China	EF437147	EF437137	PQ143666	EF437106	PQ143851	PQ143746	EF437123
Semiaquilegia danxiashanensis L.Wu, J.J.Zhou, Qiang Zhang & W.S.Deng						MK888994			MK888986
Semiaquilegia guangxiensis Yan Liu & Y.S.Huang			NC_057495	NC_057495	NC_057495	KY283995	NC_057495		KY283983
Thalictrum actaeifolium Siebold & Zucc.			JX258329		JX258544	JX573432		JX258436	JX233660
Thalictrum acutifolium (Hand.-Mazz.) B.Boivin	Sanxia Expedition 1637 (PE)	China	PQ143414	PQ143490	PQ143667	PQ143572	PQ143852	PQ143747	
Thalictrum alpinum L.			JX258333	JN895143	JX258548	JX573436		JX258440	JX233664
Thalictrum amurense Maxim.	Kharkevich S. et al. *s.n.* (PE)	Russia	PQ143415	PQ143491	PQ143668	PQ143573	PQ143853	PQ143748	PQ144403
Thalictrum aquilegiifolium L.			JX258335	KU213057	JX258550	JX573438		JX258442	JX233666
Thalictrum arkansanum B.Boivin			JX258338		JX258553	JX573441		JX258445	JX233669
Thalictrum arsenii B.Boivin			JX258339		JX258554	JX573442		JX258446	JX233670
Thalictrum atriplex Finet & Gagnep.			JX258424		JX258638	JX573522		JX258530	JX233759
Thalictrum baicalense Turcz. ex Regel			JX258341	LC617901	JX258555			JX258448	JX233672
Thalictrum brevisericeum W.T.Wang & S.H.Wang	Wang W. et al. SX2005135 (PE)	China	PQ143416	PQ143492	PQ143669	PQ143574	PQ143854	PQ143749	PQ144404
Thalictrum calabricum Spreng.			JX258342		JX258556	JX573443		JX258450	JX233674
Thalictrum calcicola T.Shimizu			JX258429		JX258644	JX573528		JX258537	JX233766
Thalictrum chelidonii DC.	Chen Z.D. et al. CMTTE 270 (PE)	China	PQ143417	PQ143493	PQ143670	PQ143575	PQ143855	PQ143750	PQ144405
Thalictrum cirrhosum H.Lév.			NC_061927	NC_061927	JX258640	JX573524	NC_061927	JX258533	JX233762
Thalictrum clavatum DC.			JX258343		JX258557	JX573444		JX258451	JX233675
Thalictrum confine Fernald			JX258344	MG221096	JX258558	JX573445		JX258452	JX233676
Thalictrum cooleyi H.E.Ahles			MT427986		MT427938	MT428020		MT427970	JF742136
Thalictrum coreanum H.Lév.			JX258346	KM206568	JX258560	JX573447	KM206568	JX258454	JX233678
Thalictrum coriaceum (Britton) Small			JX258348		JX258562	JX573449		JX258456	JX233680
Thalictrum cuernavacanum Rose			MT427987		MT427939	MT428021		MT427971	

Thalictrum cultratum Wall.			JX258426	MN492781	JX258641	JX573525		JX258534	JX233763
Thalictrum dasycarpum Fisch., C.A.Mey. & Avé-Lall.			JX258350	KJ593127	JX258564	JX573451	MT580005	JX258458	JX233682
Thalictrum decipiens B.Boivin			JX258351		JX258565	JX573452		JX258459	JX233683
Thalictrum delavayi Franch.	Sichuan's Liangshan Expedition 531 (PE)	China	PQ143418	PQ143494	PQ143671	PQ143576	PQ143856	PQ143751	PQ144406
Thalictrum diffusiflorum C.Marquand & Airy Shaw	Chen Z.D. et al. 20110804143 (PE)	China	PQ143419	PQ143495	PQ143672	PQ143577	PQ143857	PQ143752	PQ144407
Thalictrum dioicum L.			JX258354	HQ593464	JX258568	JX573455		JX258462	JX233686
Thalictrum elegans Wall. ex Royle			JX258355		JX258569	JX573456		JX258463	JX233687
Thalictrum fargesii Franch. ex Finet & Gagnep.			JX258356		JX258570	JX573457		JX258464	JX233688
Thalictrum fendleri Engelm. ex A.Gray			JX258358	OL537969	JX258572	JX573459		JX258466	JX233690
Thalictrum finetii B.Boivin			JX258427		JX258642	JX573526		JX258535	JX233764
Thalictrum flavum L.	Tichomirov V. et al. *s.n.* (PE)	Russia	PQ143420	PQ143496	PQ143673	PQ143578	PQ143858	PQ143753	PQ144408
Thalictrum foeniculaceum Bunge			JX258359	NC_053570	JX258573	JX573460	NC_053570	JX258467	JX233691
Thalictrum foetidum L.	Sichuan's Liangshan Expedition 129 (PE)	China	PQ143421	PQ143497	PQ143674	PQ143579	PQ143859	PQ143754	PQ144409
Thalictrum foliolosum DC.			JX258361	NC_058920	JX258575	JX573462	NC_058920	JX258469	JX233693
Thalictrum fortunei S.Moore	Liu M. A11025 (PE)	China	PQ143422	PQ143498	PQ143675	PQ143580	PQ143860	PQ143755	PQ144410
Thalictrum galeottii Lecoy.			JX258362		JX258576	JX573463		JX258470	JX233694
Thalictrum gibbosum Lecoy.			JX258363		JX258577	JX573464		JX258471	JX233695
Thalictrum grandiflorum Maxim.			JX258364		JX258578	JX573465		JX258472	JX233696
Thalictrum guatemalense C.DC. & Rose			JX258366		JX258580	JX573467		JX258474	JX233698
Thalictrum hamatum Maxim.	Sichuan's Liangshan Expedition 260 (PE)	China	PQ143423	PQ143499	PQ143676	PQ143581	PQ143861	PQ143756	PQ144411
Thalictrum heliophilum Wilken & DeMott			MT427991		MT427943	MT428025		MT427974	JF742154
Thalictrum henricksonii M.C.Johnst.			MT427992		MT427944	MT428026		MT427975	JF742155
Thalictrum hernandezii Tausch			JX258367		JX258581	JX573468		JX258475	JX233699
Thalictrum ichangense Lecoy. ex Oliv.	Sanxia Expedition 1413 (PE)	China	PQ143424	PQ143500	PQ143677	PQ143582	PQ143862	PQ143757	
Thalictrum integrilobum Maxim.			LC632483	LC632489					
Thalictrum isopyroides C.A.Mey.	Gu J. 03 (PE)	China	PQ143425	PQ143501	PQ143678	PQ143583	PQ143863	PQ143758	PQ144412
Thalictrum javanicum Blume	Wang W. 67 (PE)	China	AY954496	DQ478615	PQ143679	EF437107	PQ143864	PQ143759	EF437124
Thalictrum kiusianum Nakai			MT427994		MT427946	MT428028		MT427977	JF742159

Thalictrum lankesteri Standl.			JX258369		JX258583	JX573470		JX258477	JX233701
Thalictrum laxum Ulbr.	Chen Z.D. 961193 (PE)	China	PQ143426	PQ143502	PQ143680	PQ143584	PQ143865	PQ143760	PQ144413
Thalictrum lecoyeri Franch.			JX258428		JX258643	JX573527		JX258536	JX233765
Thalictrum leuconotum Franch.			JX258370		JX258584	JX573471		JX258478	JX233702
Thalictrum lucidum L.			JX258371		JX258585	JX573472		JX258479	JX233703
Thalictrum macrocarpum Gren.			JX258372		JX258586	JX573473		JX258480	JX233704
Thalictrum macrostylum Small & A.Heller			MT427996		MT427948	MT428030		MT427979	EU438837
Thalictrum microgynum Lecoy. ex Oliv.	Wang W. 25 (PE)	China	PQ143427	PQ143503	PQ143681	PQ143585	PQ143866	PQ143761	PQ144414
Thalictrum minus L.	Sanxia Expedition 2489 (PE)	China	PQ143428	PQ143504	PQ143682	PQ143586	PQ143867	PQ143762	PQ144415
Thalictrum myriophyllum Ohwi			JX258374		JX258588	JX573474		JX258482	JX233706
Thalictrum occidentale A.Gray	Chen Z.D. & Kong H.Z. CL097 (PE)	U.S.A.	PQ143429	PQ143505	PQ143683	PQ143587	PQ143868	PQ143763	PQ144416
Thalictrum omeiense W.T.Wang & S.H.Wang			JX258377		JX258591	JX573477		JX258485	JX233709
Thalictrum osmorhizoides Nakai						JQ691523			JX233710
Thalictrum peltatum DC.			JX258378		JX258592	JX573478		JX258486	JX233711
Thalictrum petaloideum L.			JX258379	MK253449	JX258593	JX573479	MK253449	JX258487	JX233712
Thalictrum pinnatum S.Watson			JX258381		JX258595	JX573481		JX258489	JX233714
Thalictrum podocarpum Kunth ex DC.			MT427997		MT427949	MT428031		MT427980	JF742169
Thalictrum polycarpum (Torr.) S.Watson			JX258383		JX258597	JX573483		JX258491	JX233716
Thalictrum polygamum Muhl. ex DC.	Halse R. R. 3583 (PE)	U.S.A.	PQ143430	PQ143506		PQ143588	PQ143869	PQ143764	PQ144433
Thalictrum pringlei S.Watson			JX258384		JX258598	JX573484		JX258492	JX233717
Thalictrum przewalskii Maxim.	Hong Y.P. 99116 (PE)	China	PQ143431	PQ143507	PQ143684	PQ143589	PQ143870	PQ143765	PQ144417
Thalictrum pubescens Pursh			JX258388	HQ593465	JX258602	JX573488		JX258496	JX233721
Thalictrum pubigerum Benth.			JX258389		JX258603	JX573489		JX258497	JX233722
Thalictrum punctatum H.Lév.			JX258390		JX258604	JQ691528		JX258498	JX233723
Thalictrum ramosum B.Boivin	Bailongjiang Expedition 1311 (PE)	China	PQ143432	PQ143508	PQ143685	PQ143590	PQ143871	PQ143766	PQ144418
Thalictrum reniforme Wall.	Chen Z.D. et al. CMTTE751 (PE)	China	PQ143433	PQ143509	PQ143686	PQ143591	PQ143872	PQ143767	PQ144419
Thalictrum reticulatum Franch.	Sichuan's Liangshan Expedition 755 (PE)	China	PQ143434	PQ143510	PQ143687	PQ143592	PQ143873	PQ143768	PQ144420
Thalictrum revolutum Le Lièvre			JX258392	MK520749	JX258606	JX573491		JX258500	JX233726
Thalictrum rhynchocarpum Quart.-Dill. & A.Rich.			JX258394	ON982101	JX258608	JX573493		JX258502	JX233728

Thalictrum robustum Maxim.	Wang W. 38 (PE)	China	EF437148	EF437138	PQ143688	EF437108	PQ143874	PQ143769	EF437125
Thalictrum rochebruneanum Franch. & Sav.			JX258395		JX258609	JX573494		JX258503	JX233729
Thalictrum rostellatum Hook.f. & Thomson			JX258397		JX258611	JX573496		JX258505	JX233731
Thalictrum rotundifolium DC.			JX258398		JX258612	JX573497		JX258506	JX233732
Thalictrum rubescens Ohwi			JX258399		JX258613	JX573498			JX233733
Thalictrum rutifolium Hook.f. & Thomson			JX258400		JX258614	JX573499		JX258507	JX233734
Thalictrum sachalinense Lecoy.	Ohashi H. et al. 99141 (PE)	Japan	PQ143435	PQ143511	PQ143689	PQ143593	PQ143875	PQ143770	PQ144421
Thalictrum saniculiforme DC.	Wakabayashi M. et al. 97-20035 (PE)	Nepal	PQ143436			PQ143594			
Thalictrum sekimotoanum Honda	Furuse M. 13660 (PE)	Japan	PQ143437			PQ143595			PQ144422
Thalictrum simplex L.	Hong Y.P. H539 (PE)	China	PQ143438	PQ143512	PQ143690	PQ143596	PQ143876	PQ143771	PQ144423
Thalictrum smithii B.Boivin			JX258404		JX258618	JX573503		JX258511	JX233738
Thalictrum sp.[1]	Chen Z.D. et al. CMTTE395 (PE)	China	PQ143439	PQ143513	PQ143691	PQ143597	PQ143877	PQ143772	
Thalictrum sp.[2]	Yu S.Y. et al. *s.n.* (PE)	China	PQ143440	PQ143514	PQ143692	PQ143598	PQ143878	PQ143773	PQ144424
Thalictrum sparsiflorum Turcz. ex Fisch. & C.A.Mey.			JX258405		JX258619	JX573504		JX258512	JX233739
Thalictrum squamiferum Lecoy.			JX258407		JX258621	JX573505		JX258514	JX233741
Thalictrum squarrosum Stephan ex Willd.			JX258408		JX258622	JX573506		JX258515	JX573506
Thalictrum strigillosum Hemsl.			JX258409		JX258623	JX573507		JX258516	JX233744
Thalictrum tenue Franch.			JX258410	MK253448	JX258624	JX573508	MK253448	JX258517	JX233745
Thalictrum texanum (E.Hall ex A.Gray) Small			JX258412		JX258626	JX573510		JX258519	JX233747
Thalictrum thalictroides (L.) A.J.Eames & B.Boivin			EU053924	KU213059	JX258627	JX573511	MH092834	NC_039433	JX233748
Thalictrum trichopus Franch.			JX258414		JX258628	JX573512		JX258520	JX233749
Thalictrum tripeltiferum B.Boivin			MT427999		MT427951	MT428033		MT427982	
Thalictrum tuberiferum Maxim.	Oh B.U. et al. 120611-004 (PE)	Korea	PQ143441	PQ143515	PQ143693	PQ143599	PQ143879		PQ144425
Thalictrum tuberosum L.			JX258416		JX258630	JX573514		JX258522	JX233751
Thalictrum uchiyamae Nakai			JX258417		JX258631	JX573515		JX258523	
Thalictrum uncatum Maxim.			JX258418	MN492785	JX258632	JX573516		JX258524	
Thalictrum uncinulatum Franch.	Wang W. 27 (PE)	China	PQ143442	PQ143516	PQ143694	PQ143600	PQ143880	PQ143774	PQ144426
Thalictrum urbaini Hayata	Chen Z.D. et al. 20110214 (PE)	China	PQ143443	PQ143517	PQ143695	PQ143601	PQ143881	PQ143775	PQ144427

Thalictrum venulosum Trel.			JX258421	MK520750	JX258635	JX573519		JX258527	JX233756
Thalictrum virgatum Hook.f. & Thomson	Chen Z.D. et al. CMTTE611 (PE)	China	PQ143444	PQ143518	PQ143696	PQ143602	PQ143882	PQ143776	PQ144428
Thalictrum wangii B.Boivin	Xie L. & Mao J.F. XM138 (PE)	China				PQ143603	PQ143883	PQ143777	PQ144429
Thalictrum zernyi Ulbr.			MT428000		MT427952	MT428034		MT427983	JF742193
Urophysa henryi (Oliv.) Ulbr.	Wang W. 96 (PE)	China	EF437149	EF437139		EF437109	PQ143884	PQ143778	EF437126
Urophysa rockii Ulbr.	Zhang L. 200910 (PE)	China	PQ143445	PQ143519	PQ143697	PQ143604	PQ143885	PQ143779	PQ144430
**Outgroups**									
Coptis chinensis Franch.			AY954497	DQ478614	NC_036485	EF437110	NC_036485	NC_036485	JF423949
Coptis trifolia (L.) Salisb.	Chen X.Q. et al. 1299 (PE)	U.S.A.	AB163771	AB695565	PQ143648	AB159542	PQ143827	PQ143781	AB695610
Xanthorhiza simplicissima Marshall	Liu Y. *s.n.* (PE)	U.S.A.	L12669	AB069848	PQ143605	EF437111	HE651098	PQ143780	KR697583
Calathodes oxycarpa Sprague			NC_041475	NC_041475	NC_041475	NC_041475	NC_041475	NC_041475	HQ440197

TABLE S2. *Primers used for amplification and sequencing in this study*

Locus	Primer	Sequence (5'-3')	Reference
rbcL	1F	ATGTCACCACAAACAGAAAC	Chen *et al*. (1998)
	1494R	GATTGGGCCGAGTTAATTAC	Chen *et al*. (1998)
matK	mF2	AAACAATCTTMTCATTTACG	Wang *et al*. (2007)
	mR2	AARGGATCCTTGAACAMCCA	Wang *et al*. (2007)
ndhA	x1	GCYCAATCWATTAGTTATGAAATACC	Shaw *et al*. (2007)
	x2	GGTTGACGCCAMARATTCCA	Shaw *et al*. (2007)
trnL-F	c	CGAAATCGGTAGACGCTACG	Taberlet *et al*. (1991)
	d	GGGGATAGAGGGACTTGAAC	Taberlet *et al*. (1991)
	e	GGTTCAAGTCCCTCTATCCC	Taberlet *et al*. (1991)
	f	ATTTGAACTGGTGACACGAG	Taberlet *et al*. (1991)
atpB-rbcL	atpB-S385R	GCGCAGATCTATGAATAGGAGACGT	Hoot *et al*. (1995)
	rbcL-1R	GAATCCAACACTTGCTTTAGTCTCT	Hoot *et al*. (1995)
rpl32-trnL	trnL$^{(UAG)}$	CTGCTTCCTAAGAGCAGCGT	Shaw *et al*. (2007)
	rpL32-F	CAGTTCCAAAAAAACGTACTTC	Shaw *et al*. (2007)
ITS	ITS-A	GGAAGGAGAAGTCGTAACAAGG	Blattner (1999)
	ITS-B	CTTTTCCTCCGCTTATTGATATG	Blattner (1999)
	ITS-C	GCAATTCACACCAAGTATCGC	Blattner (1999)
	ITS-D	CTCTCGGCAACGGATATCTCG	Blattner (1999)

TABLE S3. *Comparison of four clock models in BEAST analyses via Bayes factors*

Clock model	Marginal likelihood	Strict	Exponential	Lognormal	Random
Strict	-37103.38	-	-91.5	-143.7	92.84
Exponential	-37057.63	91.5	-	-52.2	184.34
**Lognormal**	**-37031.53**	**143.7**	**52.2**	**-**	**236.54**
Random	-37149.8	-92.84	-184.34	-236.54	-

2*ln* Bayes factor (BF) was calculated by marginal likelihoods derived from path sampling (PS) implemented in BEAST. 2*ln* BF >2.00 represents positive evidence, >6.00 represents strong evidence, and >10.00 represents very strong evidence (Kass and Raftery, 1995). The value in bold indicates the best-fit clock model.

TABLE S4. *Comparison of two tree prior processes in BEAST analyses via Bayes factors*

Tree prior	Marginal likelihood	Yule	Birth-death
Yule	-37033.52	-	-3.98
**Birth-death**	**-37031.53**	**3.98**	**-**

2*ln* Bayes factor (BF) was calculated by marginal likelihoods derived from path sampling (PS) implemented in BEAST. 2*ln* BF >2.00 represents positive evidence, >6.00 represents strong evidence, and >10.00 represents very strong evidence (Kass and Raftery, 1995). The value in bold indicates the best-fit tree prior.

TABLE S5. *Comparison of the fit of different models of biogeographical range evolution and model-specific estimates for the different parameters*

Model	Ln*L*	*n*	*d*	*e*	*j*	AICc	AICc_wt
DEC	-368.4	2	0.0110	7.30E-03	0	740.9	3.80E-17
**DEC + *j***	**-352.2**	**3**	**0.0074**	**1.00E-12**	**0.014**	**710.5**	**1.50E-10**
DIVALIKE	-379.4	2	0.0120	4.50E-09	0	762.9	6.20E-22
DIVALIKE + *j*	-362.8	3	0.0083	1.00E-12	0.014	731.7	3.60E-15

AICc, corrected Akaike Information Criterion; AICc_wt, weighted AICc; d, dispersal rate; e, extinction rate; j, relative per-event weight of jump dispersal; Ln*L*, log-likelihood; n, number of parameters. The value in bold indicates the best-fit model.

TABLE S6. *Dispersal multiplier matrix used in ancestral range reconstruction*

Regions	A	B	C	D	E	F	G	H	I
0–6 Ma									
A	1	1	0.5	0.5	0.1	0.1	0.5	0.5	0.1
B		1	1	1	0.5	0.5	0.1	0.5	0.1
C			1	1	0.5	0.1	0.1	1	0.1
D				1	0.5	0.1	0.1	1	0.1
E					1	0.1	0.1	0.5	0.01
F						1	0.1	0.1	0.5
G							1	0.1	0.01
H								1	0.1
I									1
6–35 Ma									
A	1	1	0.5	0.5	0.5	0.5	0.5	0.5	0.01
B		1	1	1	0.5	1	0.1	0.5	0.01
C			1	1	0.5	0.5	0.1	1	0.01
D				1	1	0.5	0.1	1	0.01
E					1	0.5	0.1	1	0.01
F						1	0.1	0.5	0.1
G							1	0.1	0.01
H								1	0.01
I									1
> 35 Ma									
A	1	0.1	0.1	0.1	0.1	0.5	0.5	0.1	0.01
B		1	1	1	1	0.5	0.1	0.5	0.01
C			1	1	0.5	0.1	0.1	1	0.01
D				1	1	0.1	0.1	1	0.01
E					1	0.1	0.1	0.5	0.01
F						1	0.1	0.1	0.01
G							1	0.1	0.01
H								1	0.01
I									1

Time slices and dispersal probabilities between regions are modified from Ebersbach *et al*. (2017). A, Europe; B, North Asia; C, QTP; D, East Asia; E, Japan; F, North America; G, Africa; H, Tropical Asia; I, South America.

TABLE S7. *Species, designated terminal clade, and proportion of the number of extant species sampled per terminal in BAMM and GeoSSE analyses*

Species	Terminal	Proportion sampled of terminal
Aquilegia amurensis	*Aquilegia*	0.08
Aquilegia barbaricina	*Aquilegia*	0.08
Aquilegia canadensis	*Aquilegia*	0.08
Aquilegia einseleana	*Aquilegia*	0.08
Aquilegia fragrans	*Aquilegia*	0.08
Aquilegia oxysepala	*Aquilegia*	0.08
Thalictrum actaeifolium	*Thalictrum*	0.51
Thalictrum acutifolium	*Thalictrum*	0.51
Thalictrum amurense	*Thalictrum*	0.51
Thalictrum arkansanum	*Thalictrum*	0.51
Thalictrum arsenii	*Thalictrum*	0.51
Thalictrum atriplex	*Thalictrum*	0.51
Thalictrum brevisericeum	*Thalictrum*	0.51
Thalictrum calabricum	*Thalictrum*	0.51
Thalictrum calcicola	*Thalictrum*	0.51
Thalictrum chelidonii	*Thalictrum*	0.51
Thalictrum cirrhosum	*Thalictrum*	0.51
Thalictrum clavatum	*Thalictrum*	0.51
Thalictrum confine	*Thalictrum*	0.51
Thalictrum cooleyi	*Thalictrum*	0.51
Thalictrum coreanum	*Thalictrum*	0.51
Thalictrum coriaceum	*Thalictrum*	0.51
Thalictrum cuernavacanum	*Thalictrum*	0.51
Thalictrum cultratum	*Thalictrum*	0.51
Thalictrum dasycarpum	*Thalictrum*	0.51
Thalictrum decipiens	*Thalictrum*	0.51
Thalictrum delavayi	*Thalictrum*	0.51
Thalictrum diffusiflorum	*Thalictrum*	0.51
Thalictrum dioicum	*Thalictrum*	0.51
Thalictrum elegans	*Thalictrum*	0.51
Thalictrum fargesii	*Thalictrum*	0.51
Thalictrum fendleri	*Thalictrum*	0.51
Thalictrum finetii	*Thalictrum*	0.51
Thalictrum flavum	*Thalictrum*	0.51
Thalictrum foeniculaceum	*Thalictrum*	0.51
Thalictrum foliolosum	*Thalictrum*	0.51
Thalictrum fortunei	*Thalictrum*	0.51
Thalictrum galeottii	*Thalictrum*	0.51
Thalictrum gibbosum	*Thalictrum*	0.51
Thalictrum grandiflorum	*Thalictrum*	0.51
Thalictrum guatemalense	*Thalictrum*	0.51
Thalictrum hamatum	*Thalictrum*	0.51
Thalictrum heliophilum	*Thalictrum*	0.51

Thalictrum henricksonii	*Thalictrum*	0.51
Thalictrum hernandezii	*Thalictrum*	0.51
Thalictrum ichangense	*Thalictrum*	0.51
Thalictrum integrilobum	*Thalictrum*	0.51
Thalictrum isopyroides	*Thalictrum*	0.51
Thalictrum javanicum	*Thalictrum*	0.51
Thalictrum kiusianum	*Thalictrum*	0.51
Thalictrum lankesteri	*Thalictrum*	0.51
Thalictrum laxum	*Thalictrum*	0.51
Thalictrum lecoyeri	*Thalictrum*	0.51
Thalictrum leuconotum	*Thalictrum*	0.51
Thalictrum lucidum	*Thalictrum*	0.51
Thalictrum macrocarpum	*Thalictrum*	0.51
Thalictrum macrostylum	*Thalictrum*	0.51
Thalictrum microgynum	*Thalictrum*	0.51
Thalictrum myriophyllum	*Thalictrum*	0.51
Thalictrum occidentale	*Thalictrum*	0.51
Thalictrum omeiense	*Thalictrum*	0.51
Thalictrum osmorhizoides	*Thalictrum*	0.51
Thalictrum peltatum	*Thalictrum*	0.51
Thalictrum petaloideum	*Thalictrum*	0.51
Thalictrum pinnatum	*Thalictrum*	0.51
Thalictrum podocarpum	*Thalictrum*	0.51
Thalictrum polycarpum	*Thalictrum*	0.51
Thalictrum polygamum	*Thalictrum*	0.51
Thalictrum pringlei	*Thalictrum*	0.51
Thalictrum przewalskii	*Thalictrum*	0.51
Thalictrum pubescens	*Thalictrum*	0.51
Thalictrum pubigerum	*Thalictrum*	0.51
Thalictrum punctatum	*Thalictrum*	0.51
Thalictrum ramosum	*Thalictrum*	0.51
Thalictrum reniforme	*Thalictrum*	0.51
Thalictrum reticulatum	*Thalictrum*	0.51
Thalictrum revolutum	*Thalictrum*	0.51
Thalictrum rhynchocarpum	*Thalictrum*	0.51
Thalictrum robustum	*Thalictrum*	0.51
Thalictrum rochebruneanum	*Thalictrum*	0.51
Thalictrum rostellatum	*Thalictrum*	0.51
Thalictrum rotundifolium	*Thalictrum*	0.51
Thalictrum rubescens	*Thalictrum*	0.51
Thalictrum rutifolium	*Thalictrum*	0.51
Thalictrum sachalinense	*Thalictrum*	0.51
Thalictrum saniculiforme	*Thalictrum*	0.51
Thalictrum sekimotoanum	*Thalictrum*	0.51
Thalictrum smithii	*Thalictrum*	0.51
Thalictrum sparsiflorum	*Thalictrum*	0.51
Thalictrum sp.[1]	*Thalictrum*	0.51
Thalictrum sp.[2]	*Thalictrum*	0.51

Thalictrum squamiferum	*Thalictrum*	0.51
Thalictrum squarrosum	*Thalictrum*	0.51
Thalictrum strigillosum	*Thalictrum*	0.51
Thalictrum tenue	*Thalictrum*	0.51
Thalictrum texanum	*Thalictrum*	0.51
Thalictrum thalictroides	*Thalictrum*	0.51
Thalictrum trichopus	*Thalictrum*	0.51
Thalictrum tripeltiferum	*Thalictrum*	0.51
Thalictrum tuberiferum	*Thalictrum*	0.51
Thalictrum tuberosum	*Thalictrum*	0.51
Thalictrum uchiyamae	*Thalictrum*	0.51
Thalictrum uncatum	*Thalictrum*	0.51
Thalictrum uncinulatum	*Thalictrum*	0.51
Thalictrum urbaini	*Thalictrum*	0.51
Thalictrum venulosum	*Thalictrum*	0.51
Thalictrum virgatum	*Thalictrum*	0.51
Thalictrum wangii	*Thalictrum*	0.51
Thalictrum zernyi	*Thalictrum*	0.51
Dichocarpum adiantifolium	*Dichocarpum*	0.80
Dichocarpum arisanense	*Dichocarpum*	0.80
Dichocarpum auriculatum	*Dichocarpum*	0.80
Dichocarpum basilare	*Dichocarpum*	0.80
Dichocarpum carinatum	*Dichocarpum*	0.80
Dichocarpum dalzielii	*Dichocarpum*	0.80
Dichocarpum fargesii	*Dichocarpum*	0.80
Dichocarpum franchetii	*Dichocarpum*	0.80
Dichocarpum hypoglaucum	*Dichocarpum*	0.80
Dichocarpum nipponicum	*Dichocarpum*	0.80
Dichocarpum sarmentosum	*Dichocarpum*	0.80
Dichocarpum sp.	*Dichocarpum*	0.80
Dichocarpum stoloniferum	*Dichocarpum*	0.80
Dichocarpum sutchuenense	*Dichocarpum*	0.80
Dichocarpum trachyspermum	*Dichocarpum*	0.80
Dichocarpum trifoliolatum	*Dichocarpum*	0.80
Enemion raddeanum	*Enemion*	0.75
Enemion biternatum	*Enemion*	0.75
Enemion hallii	*Enemion*	0.75
Enemion savilei	*Enemion*	0.75
Enemion stipitatum	*Enemion*	0.75
Isopyrum manshuricum	*Isopyrum*	0.75
Isopyrum thalictroides	*Isopyrum*	0.75
Leptopyrum fumarioides	*Leptopyrum*	1.00
Paraquilegia anemonoides	*Paraquilegia*	0.75
Paraquilegia microphylla	*Paraquilegia*	0.75
Paraquilegia gangotriana	*Paraquilegia*	0.75
Paraquilegia caespitosa	*Paraquilegia*	0.75
Paraquilegia scabrifolia	*Paraquilegia*	0.75
Paraquilegia uniflora	*Paraquilegia*	0.75

Paropyrum anemonoides	*Paropyrum*	1.00
Semiaquilegia adoxoides	*Semiaquilegia*	0.75
Semiaquilegia guangxiensis	*Semiaquilegia*	0.75
Semiaquilegia danxiashanensis	*Semiaquilegia*	0.75
Urophysa henryi	*Urophysa*	1.00
Urophysa rockii	*Urophysa*	1.00

TABLE S8. *Comparison of the fit of different models of GeoSSE analysis*

Model	Ln*L*	AIC	ΔAIC
full	-490.71	995.42	2.00
**no λ between regions**	**-490.71**	**993.42**	**0.00**
equal λ within regions	-493.72	999.43	6.01
equal μ within regions	-490.72	993.44	0.02
equal *q* between regions	-490.72	993.44	0.02

AIC, Akaike Information Criterion; Ln*L*, log-likelihood; λ, speciation rate; μ, extinction rate; *q*, transition rate. The value in bold indicates the best-fit model.

TABLE S9. *Estimated ages for the nodes with dispersal events*

Node	Age (Ma)	95% highest posterior density	Dispersal event
1	2.93	1.49–4.84	D to E
2	14.25	9.57–19.22	D to E; D to F
3	5.92	3.18–9.51	F to A; F to D
4	22.33	17.1–28.5	D to E
5	5.41	3.41–7.59	D to C
6	15.36	9.50–22.19	D to B; D to C
7	0.5	0.11–1.04	C to B
8	7.16	4.91–10.07	D to E; D to F
9	3.83	2.37–5.6	D to E
10	0.37	0.01–1.11	E to D
11	15.04	10.91–19.96	D to A; D to C
12	8.40	5.49–11.60	D to A; D to B
13	2.14	0.97–3.69	C to D; C to G
14	2.89	1.81–4.20	C to F
15	2.28	1.49–3.43	F to I
16	0.40	0.04–1.05	I to F
17	8.34	6.16–10.58	C to D
18	2.40	0.87–4.20	C to B; C to D; C to F
19	2.45	1.30–3.72	C to D
20	3.18	2.06–4.54	C to H
21	1.78	0.82–3.02	C to D
22	5.37	3.98–7.18	C to D
23	2.01	0.68–3.74	D to C
24	4.67	3.25–6.23	C to F
25	3.16	1.51–4.95	D to B
26	3.41	1.72–5.07	D to E
27	3.83	2.01–5.78	D to A
28	4.52	3.15–5.97	D to C; D to F; D to H
29	1.94	1.19–2.89	D to F
30	1.20	0.58–1.96	D to A; D to B
31	0.43	0.13–0.88	D to B
32	0.29	0.05–0.64	B to A; B to E
33	0.52	0.19–1.02	D to C

Divergence times were inferred from the combined plastid and ITS dataset using BEAST with the optimal clock model and tree prior. Node numbers correspond to those in Fig. 2. A, Europe; B, North Asia; C, QTP; D, East Asia; E, Japan; F, North America; G, Africa; H, Tropical Asia; I, South America.

LITERATURE CITED

Blattner FR. 1999. Direct amplification of the entire ITS region from poorly preserved plant material using recombinant PCR. *Biotechniques* **27:** 1180–1186.

Chen ZD, Wang XQ, Sun HY, *et al*. 1998. Systematic position of the Rhoipteleaceae: Evidence from nucleotide sequences of *rbcL* gene. *Acta Phytotaxonomica Sinica* **36:** 1–7.

Ebersbach J, Muellner-Riehl AN, Michalak I, *et al.* 2017. In and out of the Qinghai-Tibet Plateau: Divergence time estimation and historical biogeography of the large arctic-alpine genus *Saxifraga* L. *Journal of Biogeography* **44**: 900–910.

Hoot SB. 1995. Phylogeny of the Ranunculaceae based on *atpB, rbcL* and 18S nuclear ribosomal DNA sequence data. *Plant Systematics and Evolution* **9** (Suppl.): 241–251.

Kass RE, Raftery AE. 1995. Bayes factors. *Journal of the American Statistical Association* **90**: 773–795.

Wang W, Chen ZD, Liu Y, Li RQ, Li JH. 2007. Phylogenetic and biogeographic diversification of Berberidaceae in the northern hemisphere. *Systematic Botany* **32:** 731–742.

Wang W, Li H, Chen Z. 2014. Analysis of plastid and nuclear DNA data in plant phylogenetics—evaluation and improvement. *Science China Life Sciences* **57**: 280–286.

Shaw J, Lickey EB, Schilling EE, Small RL. 2007. Comparison of whole chloroplast genome sequences to choose noncoding regions for phylogenetic studies in angiosperms: The tortoise and the hare III. *American Journal of Botany* **94:** 275–88.

Taberlet P, Gielly L, Pautou G, Bouvet J. 1991. Universal primers for amplification of three non-coding regions of chloroplast DNA. *Plant Molecular Biology* **17:** 1105–1109.

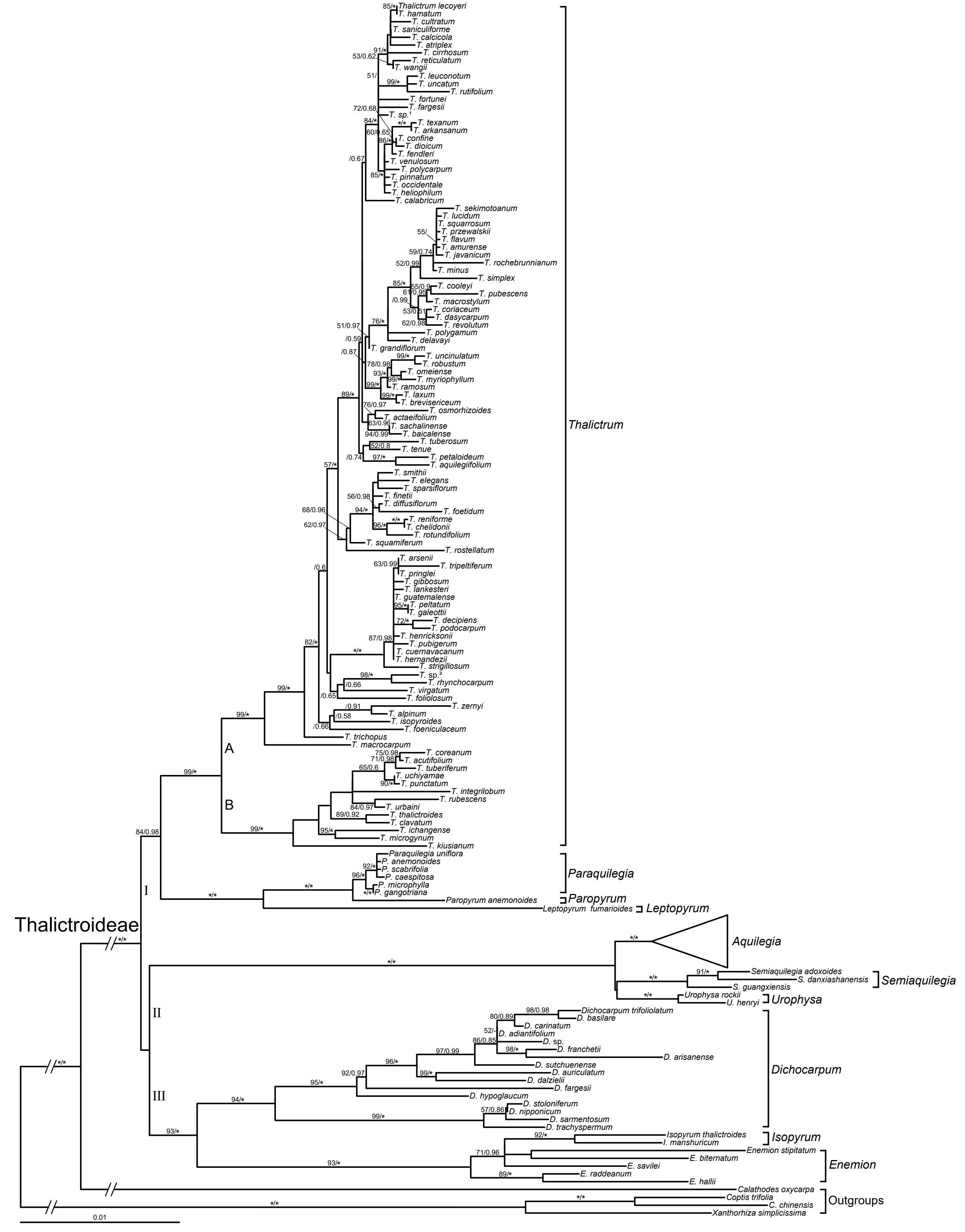

85/* Thalictrum lecoyeri
T. hamatum
T. cultratum
T. saniculiforme
T. calcicola
T. atriplex
91/* T. cirrhosum
53/0.62 T. reticulatum
T. wangii
51/
99/* T. leuconotum
T. uncatum
T. rutifolium
T. fortunei
T. fargesii
72/0.68 T. sp.[1]
84/* */* T. texanum
T. arkansanum
60/0.65 T. confine
86/* T. dioicum
T. fendleri
/0.67 T. venulosum
T. polycarpum
85/* T. pinnatum
T. occidentale
T. heliophilum
T. calabricum
T. sekimotoanum
T. lucidum
T. squarrosum
55/ T. przewalskii
T. flavum
T. amurense
59/0.74 T. javanicum
52/0.99 T. rochebrunnianum
T. minus
T. simplex
85/* 55/0.9 T. cooleyi
61/0.95 T. pubescens
/0.99 T. macrostylum
53/0.51 T. coriaceum
T. dasycarpum
62/0.98 T. revolutum
51/0.97 76/* T. polygamum
/0.59 T. delavayi
T. grandiflorum
/0.87 99/* T. uncinulatum
T. robustum
78/0.98 T. omeiense
93/* 99/* T. myriophyllum
99/* T. ramosum
99/* T. laxum
89/* T. brevisericeum
76/0.97 T. osmorhizoides
T. actaeifolium
63/0.96 T. sachalinense
94/0.99 T. baicalense
T. tuberosum
52/0.8 T. tenue
/0.74 97/* T. petaloideum
T. aquilegiifolium
57/* T. smithii
T. elegans
T. sparsiflorum
56/0.98 T. finetii
T. diffusiflorum
68/0.96 94/* T. foetidum
/ T. reniforme
62/0.97 96/* T. chelidonii
T. rotundifolium
T. squamiferum
T. rostellatum
63/0.99 T. arsenii
T. tripeltiferum
/0.6 T. pringlei
T. gibbosum
T. lankesteri
T. guatemalense
95/* T. peltatum
T. galeottii
72/* T. decipiens
T. podocarpum
87/0.98 T. henricksonii
82/* T. pubigerum
/ T. cuernavacanum
T. hernandezii
T. strigillosum
98/* T. sp.[2]
99/* /0.66 T. rhynchocarpum
T. virgatum
/0.65 T. foliolosum
/0.91 T. zernyi
T. alpinum
99/* /0.58 T. isopyroides
/0.66 T. foeniculaceum
T. trichopus
T. macrocarpum
A
75/0.98 T. coreanum
99/* 71/0.98 T. acutifolium
T. tuberiferum
65/0.6 T. uchiyamae
90/* T. punctatum
T. integrilobum
B T. rubescens
84/0.97 T. urbaini
89/0.92 T. thalictroides
T. clavatum
99/* 95/* T. ichangense
84/0.98 T. microgynum
T. kiusianum
Thalictrum
Paraquilegia uniflora
92/* P. anemonoides
P. scabrifolia
96/* P. caespitosa
P. microphylla
/ */* P. gangotriana
Paraquilegia
I */* Paropyrum anemonoides
Paropyrum
Leptopyrum fumarioides
Leptopyrum
Thalictroideae
/ */* Aquilegia
/
91/* Semiaquilegia adoxoides
/ S. danxiashanensis
S. guangxiensis
Semiaquilegia
/ Urophysa rockii
U. henryi
Urophysa
II 98/0.98 Dichocarpum trifoliolatum
80/0.89 D. basilare
D. carinatum
52/- D. adiantifolium
86/0.85 D. sp.
98/* D. franchetii
97/0.99 D. arisanense
96/* D. sutchuenense
99/* D. auriculatum
92/0.97 D. dalzielii
95/* D. fargesii
III D. hypoglaucum
94/* 57/0.86 D. stoloniferum
D. nipponicum
99/* D. sarmentosum
D. trachyspermum
Dichocarpum
93/* 92/* Isopyrum thalictroides
I. manshuricum
Isopyrum
71/0.96 Enemion stipitatum
E. biternatum
93/* E. savilei
89/* E. raddeanum
E. hallii
Enemion
/ Calathodes oxycarpa
/ Coptis trifolia
C. chinensis
Xanthorhiza simplicissima
Outgroups
0.01

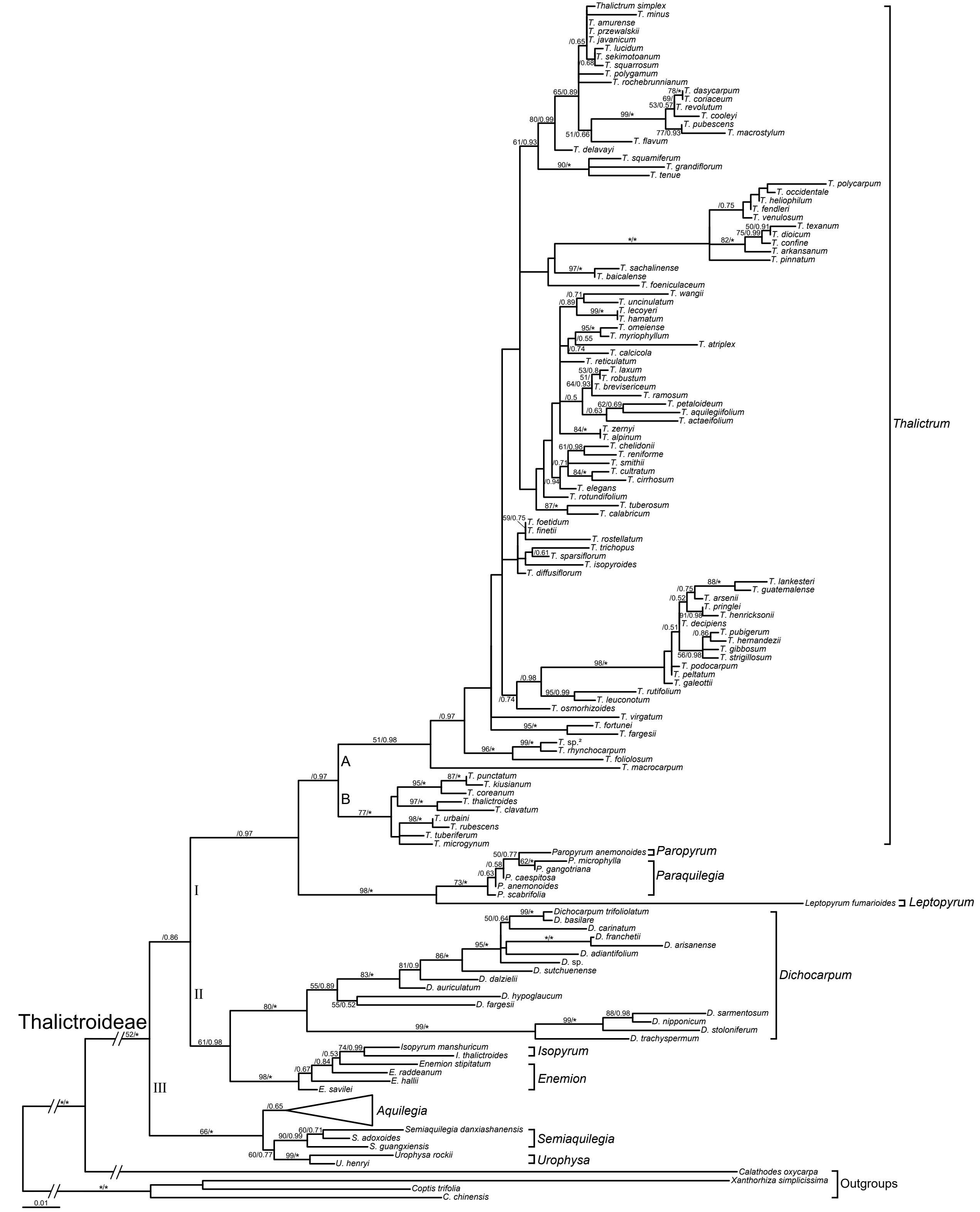
Thalictrum simplex
T. minus
T. amurense
T. przewalskii
T. javanicum
T. lucidum
T. sekimotoanum
T. squarrosum
T. polygamum
T. rochebrunnianum
T. dasycarpum
T. coriaceum
T. revolutum
T. cooleyi
T. pubescens
T. macrostylum
T. flavum
T. delavayi
T. squamiferum
T. grandiflorum
T. tenue
T. polycarpum
T. occidentale
T. heliophilum
T. fendleri
T. venulosum
T. texanum
T. dioicum
T. confine
T. arkansanum
T. pinnatum
T. sachalinense
T. baicalense
T. foeniculaceum
T. wangii
T. uncinulatum
T. lecoyeri
T. hamatum
T. omeiense
T. myriophyllum
T. atriplex
T. calcicola
T. reticulatum
T. laxum
T. robustum
T. brevisericeum
T. ramosum
T. petaloideum
T. aquilegiifolium
T. actaeifolium
T. zernyi
T. alpinum
T. chelidonii
T. reniforme
T. smithii
T. cultratum
T. cirrhosum
T. elegans
T. rotundifolium
T. tuberosum
T. calabricum
T. foetidum
T. finetii
T. rostellatum
T. trichopus
T. sparsiflorum
T. isopyroides
T. diffusiflorum
T. lankesteri
T. guatemalense
T. arsenii
T. pringlei
T. henricksonii
T. decipiens
T. pubigerum
T. hernandezii
T. gibbosum
T. strigillosum
T. podocarpum
T. peltatum
T. galeottii
T. rutifolium
T. leuconotum
T. osmorhizoides
T. virgatum
T. fortunei
T. fargesii
T. sp.²
T. rhynchocarpum
T. foliolosum
T. macrocarpum
T. punctatum
T. kiusianum
T. coreanum
T. thalictroides
T. clavatum
T. urbaini
T. rubescens
T. tuberiferum
T. microgynum
Thalictrum
A
B
Paropyrum anemonoides
Paropyrum
P. microphylla
P. gangotriana
P. caespitosa
P. anemonoides
P. scabrifolia
Paraquilegia
Leptopyrum fumarioides
Leptopyrum
Dichocarpum trifoliolatum
D. basilare
D. carinatum
D. franchetii
D. arisanense
D. adiantifolium
D. sp.
D. sutchuenense
D. dalzielii
D. auriculatum
D. hypoglaucum
D. fargesii
D. sarmentosum
D. nipponicum
D. stoloniferum
D. trachyspermum
Dichocarpum
Isopyrum manshuricum
I. thalictroides
Isopyrum
Enemion stipitatum
E. raddeanum
E. hallii
E. savilei
Enemion
Aquilegia
Semiaquilegia danxiashanensis
S. adoxoides
S. guangxiensis
Semiaquilegia
Urophysa rockii
U. henryi
Urophysa
Calathodes oxycarpa
Xanthorhiza simplicissima
Coptis trifolia
C. chinensis
Outgroups
Thalictroideae
I
II
III
0.01

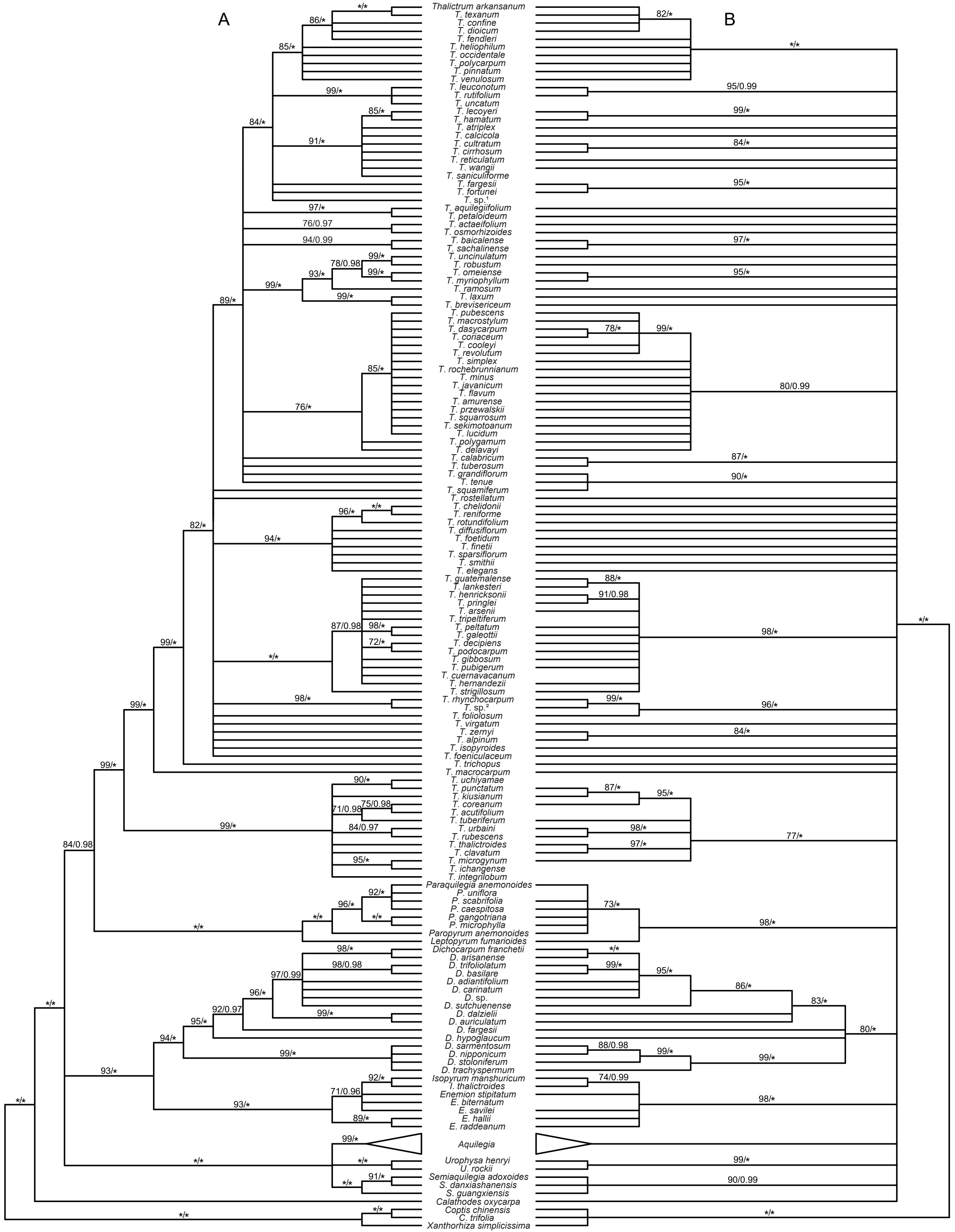
A
B
Thalictrum arkansanum
T. texanum
T. confine
T. dioicum
T. fendleri
T. heliophilum
T. occidentale
T. polycarpum
T. pinnatum
T. venulosum
T. leuconotum
T. rutifolium
T. uncatum
T. lecoyeri
T. hamatum
T. atriplex
T. calcicola
T. cultratum
T. cirrhosum
T. reticulatum
T. wangii
T. saniculiforme
T. fargesii
T. fortunei
T. sp.[1]
T. aquilegiifolium
T. petaloideum
T. actaeifolium
T. osmorhizoides
T. baicalense
T. sachalinense
T. uncinulatum
T. robustum
T. omeiense
T. myriophyllum
T. ramosum
T. laxum
T. brevisericeum
T. pubescens
T. macrostylum
T. dasycarpum
T. coriaceum
T. cooleyi
T. revolutum
T. simplex
T. rochebrunnianum
T. minus
T. javanicum
T. flavum
T. amurense
T. przewalskii
T. squarrosum
T. sekimotoanum
T. lucidum
T. polygamum
T. delavayi
T. calabricum
T. tuberosum
T. grandiflorum
T. tenue
T. squamiferum
T. rostellatum
T. chelidonii
T. reniforme
T. rotundifolium
T. diffusiflorum
T. foetidum
T. finetii
T. sparsiflorum
T. smithii
T. elegans
T. guatemalense
T. lankesteri
T. henricksonii
T. pringlei
T. arsenii
T. tripeltiferum
T. peltatum
T. galeottii
T. decipiens
T. podocarpum
T. gibbosum
T. pubigerum
T. cuernavacanum
T. hernandezii
T. strigillosum
T. rhynchocarpum
T. sp.[2]
T. foliolosum
T. virgatum
T. zernyi
T. alpinum
T. isopyroides
T. foeniculaceum
T. trichopus
T. macrocarpum
T. uchiyamae
T. punctatum
T. kiusianum
T. coreanum
T. acutifolium
T. tuberiferum
T. urbaini
T. rubescens
T. thalictroides
T. clavatum
T. microgynum
T. ichangense
T. integrilobum
Paraquilegia anemonoides
P. uniflora
P. scabrifolia
P. caespitosa
P. gangotriana
P. microphylla
Paropyrum anemonoides
Leptopyrum fumarioides
Dichocarpum franchetii
D. arisanense
D. trifoliolatum
D. basilare
D. adiantifolium
D. carinatum
D. sp.
D. sutchuenense
D. dalzielii
D. auriculatum
D. fargesii
D. hypoglaucum
D. sarmentosum
D. nipponicum
D. stoloniferum
D. trachyspermum
Isopyrum manshuricum
I. thalictroides
Enemion stipitatum
E. biternatum
E. savilei
E. hallii
E. raddeanum
Aquilegia
Urophysa henryi
U. rockii
Semiaquilegia adoxoides
S. danxiashanensis
S. guangxiensis
Calathodes oxycarpa
Coptis chinensis
C. trifolia
Xanthorhiza simplicissima

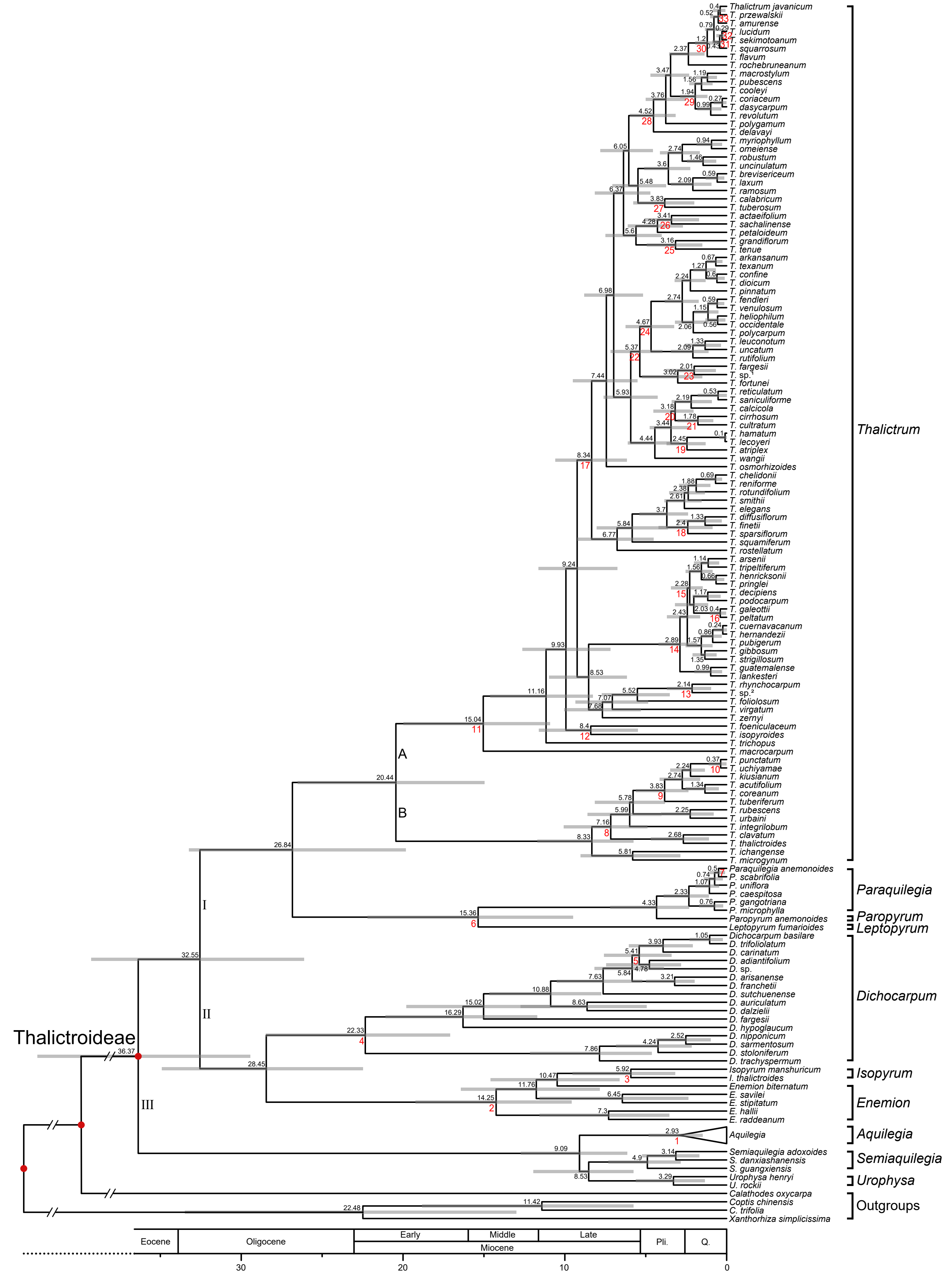

Thalictrum javanicum
T. przewalskii
T. amurense
T. lucidum
T. sekimotoanum
T. squarrosum
T. flavum
T. rochebruneanum
T. macrostylum
T. pubescens
T. cooleyi
T. coriaceum
T. dasycarpum
T. revolutum
T. polygamum
T. delavayi
T. myriophyllum
T. omeiense
T. robustum
T. uncinulatum
T. brevisericeum
T. laxum
T. ramosum
T. calabricum
T. tuberosum
T. actaeifolium
T. sachalinense
T. petaloideum
T. grandiflorum
T. tenue
T. arkansanum
T. texanum
T. confine
T. dioicum
T. pinnatum
T. fendleri
T. venulosum
T. heliophilum
T. occidentale
T. polycarpum
T. leuconotum
T. uncatum
T. rutifolium
T. fargesii
T. sp.¹
T. fortunei
T. reticulatum
T. saniculiforme
T. calcicola
T. cirrhosum
T. cultratum
T. hamatum
T. lecoyeri
T. atriplex
T. wangii
T. osmorhizoides
T. chelidonii
T. reniforme
T. rotundifolium
T. smithii
T. elegans
T. diffusiflorum
T. finetii
T. sparsiflorum
T. squamiferum
T. rostellatum
T. arsenii
T. tripeltiferum
T. henricksonii
T. pringlei
T. decipiens
T. podocarpum
T. galeottii
T. peltatum
T. cuernavacanum
T. hernandezii
T. pubigerum
T. gibbosum
T. strigillosum
T. guatemalense
T. lankesteri
T. rhynchocarpum
T. sp.²
T. foliolosum
T. virgatum
T. zernyi
T. foeniculaceum
T. isopyroides
T. trichopus
T. macrocarpum
T. punctatum
T. uchiyamae
T. kiusianum
T. acutifolium
T. coreanum
T. tuberiferum
T. rubescens
T. urbaini
T. integrilobum
T. clavatum
T. thalictroides
T. ichangense
T. microgynum
Paraquilegia anemonoides
P. scabrifolia
P. uniflora
P. caespitosa
P. gangotriana
P. microphylla
Paropyrum anemonoides
Leptopyrum fumarioides
Dichocarpum basilare
D. trifoliolatum
D. carinatum
D. adiantifolium
D. sp.
D. arisanense
D. franchetii
D. sutchuenense
D. auriculatum
D. dalzielii
D. fargesii
D. hypoglaucum
D. nipponicum
D. sarmentosum
D. stoloniferum
D. trachyspermum
Isopyrum manshuricum
I. thalictroides
Enemion biternatum
E. savilei
E. stipitatum
E. hallii
E. raddeanum
Aquilegia
Semiaquilegia adoxoides
S. danxiashanensis
S. guangxiensis
Urophysa henryi
U. rockii
Calathodes oxycarpa
Coptis chinensis
C. trifolia
Xanthorhiza simplicissima
Thalictrum
Paraquilegia
Paropyrum
Leptopyrum
Dichocarpum
Isopyrum
Enemion
Aquilegia
Semiaquilegia
Urophysa
Outgroups
Thalictroideae
A
B
I
II
III
Eocene
Oligocene
Early
Middle
Late
Miocene
Pli.
Q.
30
20
10
0

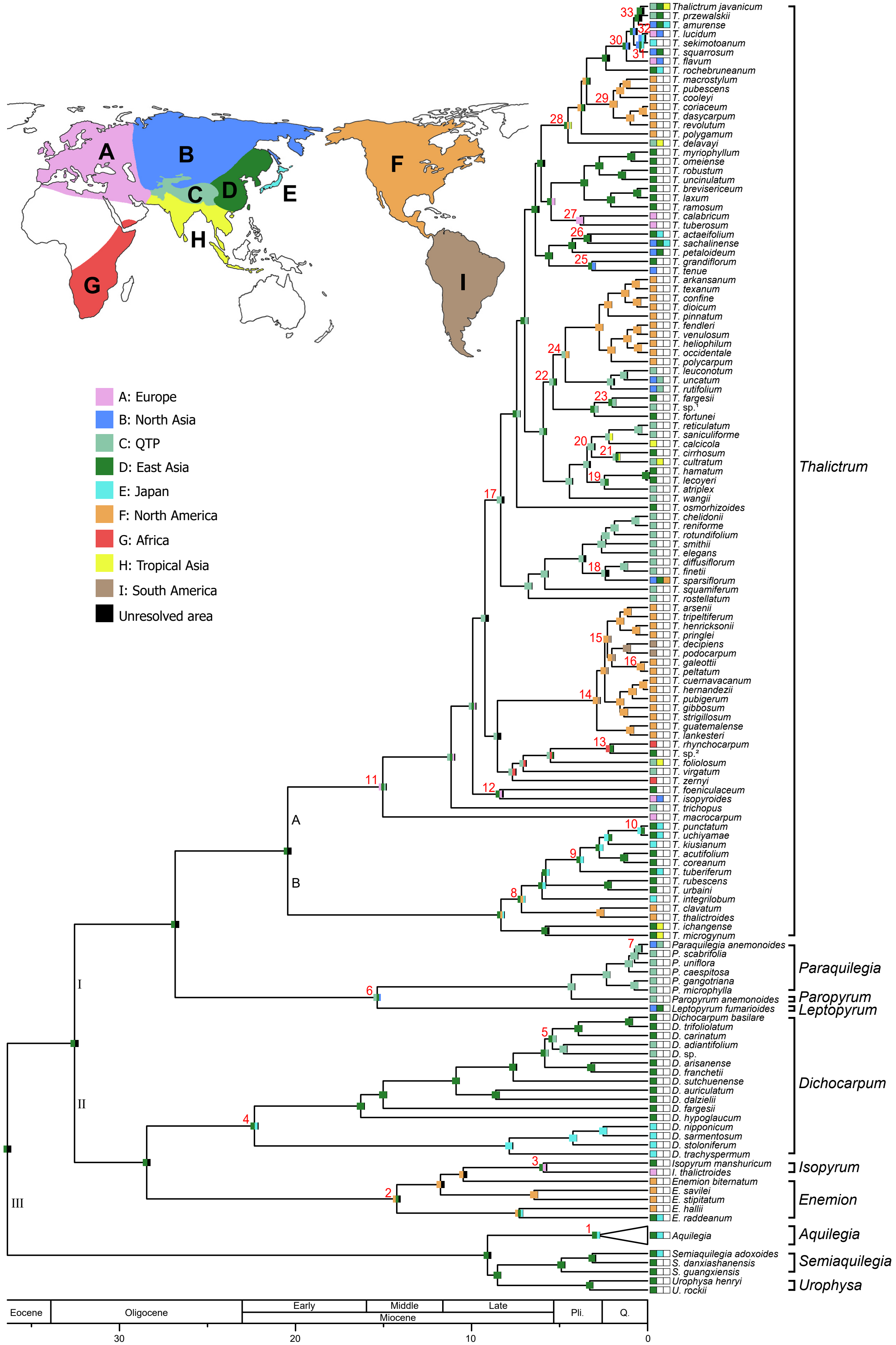

A
B
C
D
E
F
G
H
I
A: Europe
B: North Asia
C: QTP
D: East Asia
E: Japan
F: North America
G: Africa
H: Tropical Asia
I: South America
Unresolved area
Thalictrum javanicum
T. przewalskii
T. amurense
T. lucidum
T. sekimotoanum
T. squarrosum
T. flavum
T. rochebruneanum
T. macrostylum
T. pubescens
T. cooleyi
T. coriaceum
T. dasycarpum
T. revolutum
T. polygamum
T. delavayi
T. myriophyllum
T. omeiense
T. robustum
T. uncinulatum
T. brevisericeum
T. laxum
T. ramosum
T. calabricum
T. tuberosum
T. actaeifolium
T. sachalinense
T. petaloideum
T. grandiflorum
T. tenue
T. arkansanum
T. texanum
T. confine
T. dioicum
T. pinnatum
T. fendleri
T. venulosum
T. heliophilum
T. occidentale
T. polycarpum
T. leuconotum
T. uncatum
T. rutifolium
T. fargesii
T. sp.[1]
T. fortunei
T. reticulatum
T. saniculiforme
T. calcicola
T. cirrhosum
T. cultratum
T. hamatum
T. lecoyeri
T. atriplex
T. wangii
T. osmorhizoides
T. chelidonii
T. reniforme
T. rotundifolium
T. smithii
T. elegans
T. diffusiflorum
T. finetii
T. sparsiflorum
T. squamiferum
T. rostellatum
T. arsenii
T. tripeltiferum
T. henricksonii
T. pringlei
T. decipiens
T. podocarpum
T. galeottii
T. peltatum
T. cuernavacanum
T. hernandezii
T. pubigerum
T. gibbosum
T. strigillosum
T. guatemalense
T. lankesteri
T. rhynchocarpum
T. sp.[2]
T. foliolosum
T. virgatum
T. zernyi
T. foeniculaceum
T. isopyroides
T. trichopus
T. macrocarpum
T. punctatum
T. uchiyamae
T. kiusianum
T. acutifolium
T. coreanum
T. tuberiferum
T. rubescens
T. urbaini
T. integrilobum
T. clavatum
T. thalictroides
T. ichangense
T. microgynum
Thalictrum
Paraquilegia anemonoides
P. scabrifolia
P. uniflora
P. caespitosa
P. gangotriana
P. microphylla
Paraquilegia
Paropyrum anemonoides
Paropyrum
Leptopyrum fumarioides
Leptopyrum
Dichocarpum basilare
D. trifoliolatum
D. carinatum
D. adiantifolium
D. sp.
D. arisanense
D. franchetii
D. sutchuenense
D. auriculatum
D. dalzielii
D. fargesii
D. hypoglaucum
D. nipponicum
D. sarmentosum
D. stoloniferum
D. trachyspermum
Dichocarpum
Isopyrum manshuricum
I. thalictroides
Isopyrum
Enemion biternatum
E. savilei
E. stipitatum
E. hallii
E. raddeanum
Enemion
Aquilegia
Aquilegia
Semiaquilegia adoxoides
S. danxiashanensis
S. guangxiensis
Semiaquilegia
Urophysa henryi
U. rockii
Urophysa
A
B
I
II
III
Eocene
Oligocene
Early
Middle
Late
Miocene
Pli.
Q.
30
20
10
0

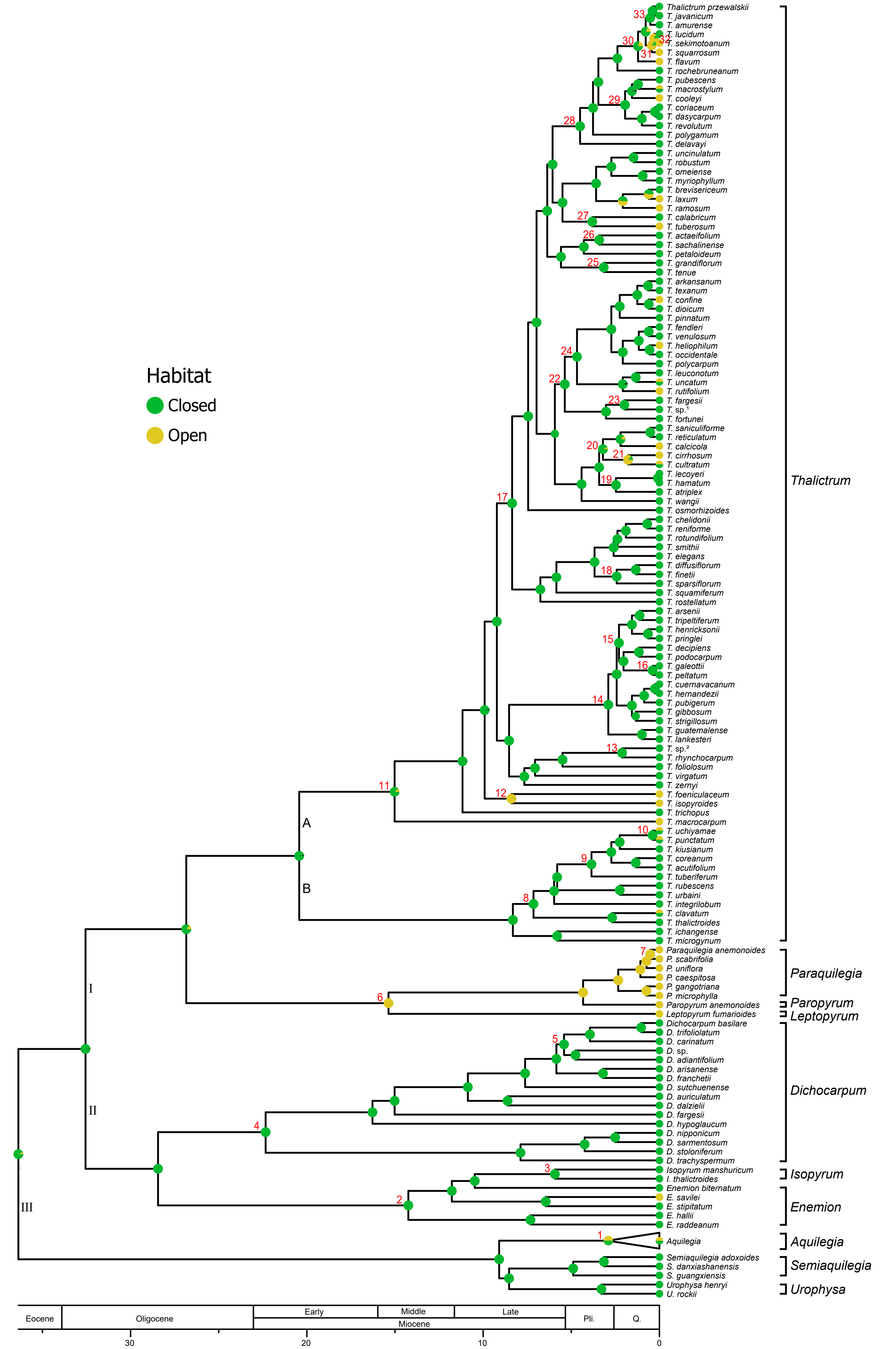
Habitat
Closed
Open
Thalictrum przewalskii
T. javanicum
T. amurense
T. lucidum
T. sekimotoanum
T. squarrosum
T. flavum
T. rochebruneanum
T. pubescens
T. macrostylum
T. cooleyi
T. coriaceum
T. dasycarpum
T. revolutum
T. polygamum
T. delavayi
T. uncinulatum
T. robustum
T. omeiense
T. myriophyllum
T. brevisericeum
T. laxum
T. ramosum
T. calabricum
T. tuberosum
T. actaeifolium
T. sachalinense
T. petaloideum
T. grandiflorum
T. tenue
T. arkansanum
T. texanum
T. confine
T. dioicum
T. pinnatum
T. fendleri
T. venulosum
T. heliophilum
T. occidentale
T. polycarpum
T. leuconotum
T. uncatum
T. rutifolium
T. fargesii
T. sp.¹
T. fortunei
T. saniculiforme
T. reticulatum
T. calcicola
T. cirrhosum
T. cultratum
T. lecoyeri
T. hamatum
T. atriplex
T. wangii
T. osmorhizoides
T. chelidonii
T. reniforme
T. rotundifolium
T. smithii
T. elegans
T. diffusiflorum
T. finetii
T. sparsiflorum
T. squamiferum
T. rostellatum
T. arsenii
T. tripeltiferum
T. henricksonii
T. pringlei
T. decipiens
T. podocarpum
T. galeottii
T. peltatum
T. cuernavacanum
T. hernandezii
T. pubigerum
T. gibbosum
T. strigillosum
T. guatemalense
T. lankesteri
T. sp.²
T. rhynchocarpum
T. foliolosum
T. virgatum
T. zernyi
T. foeniculaceum
T. isopyroides
T. trichopus
T. macrocarpum
T. uchiyamae
T. punctatum
T. kiusianum
T. coreanum
T. acutifolium
T. tuberiferum
T. rubescens
T. urbaini
T. integrilobum
T. clavatum
T. thalictroides
T. ichangense
T. microgynum
Thalictrum
Paraquilegia anemonoides
P. scabrifolia
P. uniflora
P. caespitosa
P. gangotriana
P. microphylla
Paraquilegia
Paropyrum anemonoides
Paropyrum
Leptopyrum fumarioides
Leptopyrum
Dichocarpum basilare
D. trifoliolatum
D. carinatum
D. sp.
D. adiantifolium
D. arisanense
D. franchetii
D. sutchuenense
D. auriculatum
D. dalzielii
D. fargesii
D. hypoglaucum
D. nipponicum
D. sarmentosum
D. stoloniferum
D. trachyspermum
Dichocarpum
Isopyrum manshuricum
I. thalictroides
Isopyrum
Enemion biternatum
E. savilei
E. stipitatum
E. hallii
E. raddeanum
Enemion
Aquilegia
Aquilegia
Semiaquilegia adoxoides
S. danxiashanensis
S. guangxiensis
Semiaquilegia
Urophysa henryi
U. rockii
Urophysa
A
B
I
II
III
1
2
3
4
5
6
7
8
9
10
11
12
13
14
15
16
17
18
19
20
21
22
23
24
25
26
27
28
29
30
31
32
33
Eocene
Oligocene
Early
Middle
Late
Miocene
Pli.
Q.
30
20
10
0
